# Network Controllability Reveals Key Mitigation Points for Tumor-Promoting Signaling in Tumor-Educated Platelets

**DOI:** 10.3390/ijms262110780

**Published:** 2025-11-05

**Authors:** Özge Osmanoglu, Elif Özer, Shishir K. Gupta, Katrin G. Heinze, Harald Schulze, Thomas Dandekar

**Affiliations:** 1 Functional Genomics & Systems Biology Group, Department of Bioinformatics, Biocenter, Am Hubland, University of Wuerzburg, 97074 Würzburg, Germanyshishir.gupta@cbmr.res.in (S.K.G.); 2 Department of Data Sciences, Centre of Biomedical Research, Sanjay Gandhi Post-Graduate Institute of Medical Sciences Campus, Raebareli Road, Lucknow 226014, India; 3 Rudolf Virchow Center for Integrative and Translational Bioimaging, Julius-Maximilians-Universität Würzburg (JMU), Josef-Schneider-Str. 2, 97080 Würzburg, Germany; 4 Institute of Experimental Biomedicine I, University Hospital Würzburg, Josef-Schneider-Str. 2, 97080 Würzburg, Germany; 5 BioComputing Unit, European Molecular Biology Laboratory (EMBL) Heidelberg, Meyerhofstraße 1, 69117 Heidelberg, Germany

**Keywords:** tumor-educated platelets (TEP), signaling, ITAM, P2Y12, cancer, non-small cell lung cancer (NSCLC)

## Abstract

Therapeutic strategies targeting “tumor-educated platelets” (TEPs) and platelet–tumor interactions by key signaling pathways (ITAM, P2Y12) may reduce metastasis and cancer. Using a TEP gene expression dataset originally created to study swarm intelligence-enhanced detection of lung cancer cells (GSE89843), we did perform extensive transcriptome analysis to integrate these data with directed protein–protein interactions and build a TEP-specific signaling network. We analyze network topology and controllability and identify critical and indispensable nodes, as well as high-weight, usually high-score nodes. We reconstruct (pharmacological) controllable subnetworks of TEP signaling, which we then explore for drugs targets. We found 111 upregulated and 108 downregulated genes compared to control platelets, enriched in pathways related to extracellular matrix interactions, cytoskeleton organization, immune signaling, and platelet activation. Ribosomal function, apoptosis, and immune signaling were among the downregulated processes, highlighting unique TEP profiles in non-small-cell lung cancer (NSCLC). Our integrative analysis of TEPs in NSCLC reveals key transcriptional and network-based alterations harmful for the cancer patient. Using four complementary strategies, we identified five high-confidence genes (Gene symbols always given throughout the paper), *ITGA2B*, *FLNA*, *GRB2*, *FCGR2A*, and *APP*, as central to TEP signaling. These can be targeted by FDA-approved drugs. Fostamatinib, an SYK inhibitor, emerged as the top candidate drug to disrupt ITAM-mediated platelet activation selectively; metastasis-promoting metalloprotease and cytoskeletal targets influencing adhesion were also identified. A low-dose combination therapy of fostamatinib, Aducanumab, and acetylsalicylic acid (aspirin) may control TEP effects. In conclusion, our preclinical in silico approach revealed FDA-approved drugs that allow therapeutic targeting of metastasis-promoting TEPs and target NSCLC at the same time.

## 1. Introduction

Armand Trousseau was the first (in 1865) to observe the connection between cancer and platelets [1]. This discovery was followed by many others who advanced the understanding of the relationship between platelets and cancer [2,3,4,5,6]. What is now referred to as “Trousseau’s syndrome” describes increased incidence and severity of thrombotic events with increased cancer progression, sometimes even detected before the cancer diagnosis [7,8,9,10,11,12]. However, this relationship is also observed vice versa, in which higher cancer progression and metastasis is observed with increased platelet numbers/activity [13].

The effect of tumor cells on platelets can be via activities such as promoting platelet production [4,14], changing platelet transcriptomes and proteomes [15,16,17], or by changing platelet signaling and activity (review in [18]). These are collectively designated as “platelet education”.

One of the main mechanisms of platelet education is the transfer and storage of tumor-associated factors (proteins, DNA, or RNA) into platelets [19,20,21], which are then carried to dormant tumors at distant sites, aiding angiogenesis and preparing the pre-metastatic niche [22,23].

A second mechanism of platelet education involves platelet RNA processing and production [24]. Although anucleate, platelets can still synthesize proteins in response to various signals [25]. This occurs through differential splicing and alternative splicing of pre-RNA to mRNA [26], as well as alterations directly within the gene expression profile of megakaryocytes [17,24]. Alterations in the noncoding RNAs have also been observed in platelets during cancer [27,28,29,30,31]. It is still unclear whether these changes affect platelet gene expression and contribute to their platelet education by tumor cells.

Tumor cells can activate platelets directly by molecules on the tumor cell surface [32,33,34,35] or those released by tumor cells [35,36,37], leading to tumor cell-induced platelet aggregation (TCIPA) [38], which is observed in several cancer types, including lung [37], ovarian, pancreatic, and breast cancer [39]. Indirectly, cancer cells can lead to platelet activation by interacting with other cells in the tumor microenvironment, including inducing formation of NETs [40,41] and activating the granulocytes [42].

In return, activated platelets contribute to cancer progression in multiple ways [18], promoting tumor cell proliferation by releasing growth factors, cytokines, and pro- and anti-angiogenic molecules [23,40,43,44,45,46,47], shielding them from immune responses [36,40,41,42,48,49], inducing epithelial–mesenchymal transition (EMT) [40,50], protecting them from shear stress in the bloodstream, supporting tumor cell adhesion to endothelium [40,41,51,52,53], establishing metastatic sites [32,41,42,54], and enabling tumor cell extravasation to the metastasis site [40,51,55,56,57,58,59].

The relationship between platelets and cancer can be explored as various therapeutic opportunities. Increased platelet counts are often observed in cancer patients [60], suggesting that platelets could be used for diagnostics [61], as well as to monitor cancer progression and treatment response. Beyond diagnostics, platelets can also be utilized directly for cancer treatment, either as drug delivery systems or as therapeutic targets [62]. Use of anti-platelet agents for cancer therapy can be beneficial in two ways. First, they can be utilized to target the interactions between cancer cells and platelets. The platelet–tumor crosstalk is mediated through direct cellular contact, activation of cell surface receptors, release of soluble proteins, and shedding of microparticles [40]. Targeting platelet–cancer cell interactions aim to disrupt the protective role that platelets play in shielding tumor cells from immune detection and facilitating metastasis [63,64]. By interfering with the mechanisms that cancer cells use to activate and aggregate platelets, this approach could potentially reduce the spread of circulating tumor cells, enhancing the effectiveness of existing cancer treatments.

Moreover, targeting platelet signaling pathways that contribute to cancer progression, such as those involved in inflammation and thrombosis, offers another therapeutic avenue. Drugs like acetylsalicylic acid (aspirin), which inhibits cyclooxygenase-1 (COX-1), have shown promise in reducing the risk of certain cancers, such as colorectal cancer [63,64,65,66,67]. Other anti-platelet agents such as P2Y_12_ inhibitor ticagrelor [41,50,68] and antibodies against integrin αIIbβ3 [69,70] were shown to have anti-metastatic effects. This approach may provide a broad-spectrum effect by reducing risk of thrombotic complications that arise from sustained activation of platelets [71,72,73], and by slowing down cancer progression and metastasis and thereby improving patient outcomes. However, the long-term use of anti-platelet drugs comes with an increased risk of bleeding, and their efficacy may vary depending on the cancer type and stage. Inhibiting platelet kinases may at the same time also target cancer-promoting kinases in cancer cells. Bearing these complex relationships in mind, further research is required to determine the optimal regime of anti-platelet therapies implied by our basic target options from our network analysis [74].

To identify anti-platelet therapeutic options targeting regulated platelet signaling in cancer, we performed transcriptome analysis of experimental data from a unique gene expression dataset on tumor-educated platelets, GEO dataset GSE89843 [75]. This dataset was created to study swarm intelligence-enhanced detection of non-small cell lung cancer using tumor-educated platelets [75], but the transcriptome data was not yet analyzed. Moreover, we integrated experimental information from OmniPath database [76] regarding the implied protein–protein interactions. This combination of experimental validated data allowed us to construct a TEP-specific signaling network. Network topology and controllability analysis identified key proteins essential to TEP signaling. We reconstructed controllable subnetworks and explored potential drug targets, revealing genes associated with platelet activation, immune signaling, and cytoskeleton organization. Screening these subnetworks identified several FDA-approved drugs. Fostamatinib, a SYK inhibitor controlling platelet hyperactivity [77,78], emerged again as a top candidate drug, as it controls and disrupts the wide-spread ITAM-mediated platelet activation. However, metastasis-promoting metalloprotease and cytoskeletal targets influencing adhesion were also identified as important targets. In conclusion, our approach revealed FDA-approved drugs that allow therapeutic targeting of metastasis-promoting TEPs and target NSCLC at the same time.

## 2. Results

### 2.1. RNA Profiles of Tumor-Educated Platelets in NSCLC

**Figure 1:**
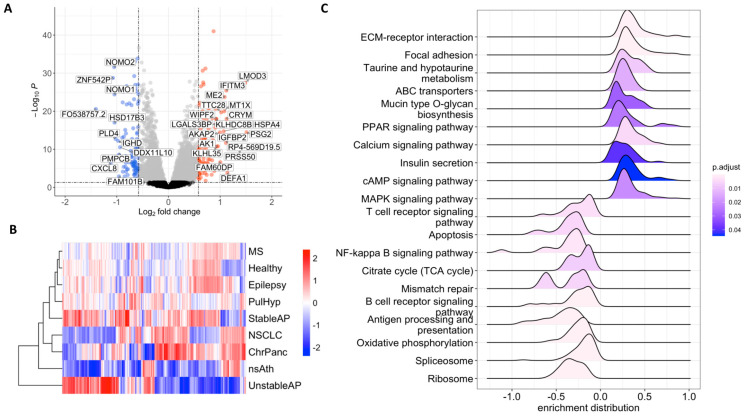
Differential Gene Expression Analysis of TEPs in NSCLC patients reveal key genes in metastasis promotion. (**A**) Top 30 upregulated (red) and downregulated (blue) genes in TEPs in NSCLC patients. (grey = Log_2_FoldChange < 0.5, black = Log_2_FoldChange < 0.5 & adj.*p*-value > 0.05 ) (**B**) Relationship between the expression of DEGs in different conditions. (**C**) Gene Set Enrichment Analysis of NSCLC-TEPs showing activated and suppressed KEGG pathways. MS: multiple sclerosis, PulHyp: pulmonary hypertension, StableAP: stable angina pectoris, NSCLC: non-small-cell lung cancer, ChrPanc: chronic pancreatitis, nsAth: non-significant atherosclerosis, UnstableAP: unstable angina pectoris.

We compared the gene expression profile in platelets from NSCLC patients to platelets from non-cancer donors. A total of 60% of the donors with no known cancer were classified as healthy, and 40% were diagnosed with inflammatory conditions (Figure 1A, Table S1). We found 111 upregulated and 108 downregulated genes in tumor-educated platelets (TEPs) compared to platelets from a non-cancer condition (Figure 1A, Datasheet S1).

Gene expression of the differentially expressed genes in chronic pancreatitis (ChrPanc) appeared to be the most similar condition to NSCLC in terms of expression profiles of the DEGs. While platelets of multiple sclerosis (MS) patients showed a profile similar to healthy patients, followed by epilepsy patients, patients with pulmonary hypertension (PulHyp), and stable angina pectoris (stableAP), DEG profiles of platelets from patients with unstable angina pectoris (unstableAP) and non-significant atherosclerosis (nsAth) appeared to be the most diverse when compared to the rest of the samples (Figure 1B).

Upregulated genes were predominantly involved in KEGG pathways associated with ECM–receptor interaction, focal adhesion, metabolism of taurine, biosynthesis of mucins, signaling pathways of calcium, PPAR, cAMP and MAPKs, and insulin secretion. Conversely, downregulated genes were enriched in immune-related pathways such as T-cell and B-cell signaling, NF-κB signaling, and antigen processing, as well as apoptosis, TCA cycle and oxidative phosphorylation, and ribosome- and spliceosome-related pathways. These findings highlight a shift in platelet function in NSCLC, favoring pro-adhesive and ECM-remodeling activities while downregulating immune-associated signaling. (Figure 1C).

Gene enrichment analysis using Gene Ontology Biological Process Terms and Reactome Pathways also highlighted similar pathways associated with apoptosis, B- and T-cell signaling, NF-KB signaling, translation and post-translational processes, and DNA repair as downregulated. Upregulation of pathways and processes associated with cell–cell or cell––ECM interaction, ECM organization, and cytoskeleton continued to be consistently observed (Figure S1, Datasheets S2–S4).

In summary, our findings indicate that TEPs exhibit increased gene expression of genes associated with cell–cell interactions, extracellular matrix (ECM) dynamics, platelet activation, and cytoskeleton organization. Conversely, genes related to ribosomal functions, apoptosis, and immune processes, particularly those involving MHC class II and B and T-cell signaling, are downregulated. These results highlight the altered cellular processes in TEPs and provide insight into their potential role in cancer.

### 2.2. Therapeutic Target Discovery Based on Transcriptome Data

**Figure 2:**
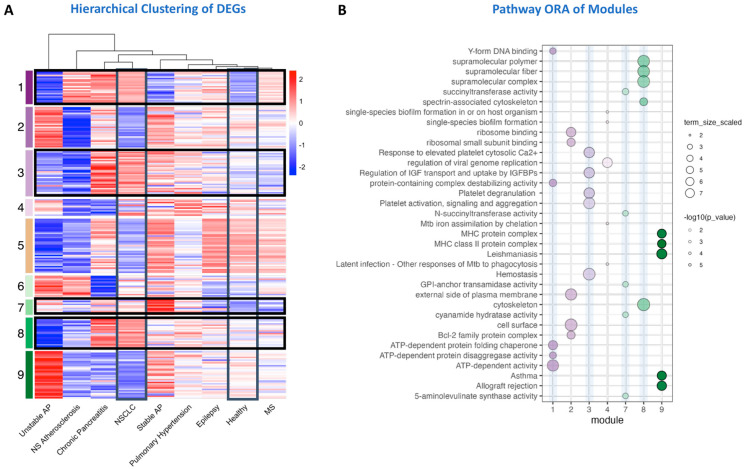
Gene Expression Profile of TEPs in NSCLC compared to the profile of control patients with other disease entities. (**A**) Hierarchical clustering of the differentially expressed genes according to disease for all different patient groups and data contained in GSE89843. Vertical black boxes mark cancer (NSCLC) and healthy control. Horizontal boxes mark protein-protein interaction networks (“modules”) with the most upregulated genes in NSCLC (modules 1, 3, 7, and 8). (**B**) Combined overrepresentation analysis of Gene Ontology (GO) molecular function. Module membership (modules ranging from 1-4 and 7-9) indicated by purple and green in different shades. In addition, we show cellular compartment, biological process as well as KEGG and REACTOME pathways in each module (vertical modules, numbered from 1 to 9 as in (**A**), module colors are matching).

Using this gene expression dataset, we employed four complementary strategies to identify potential therapeutic targets. The first two strategies are solely based on differential gene expression, while the latter two integrated network-based analyses to enhance target selection. To ensure that the regulated pathways were NSCLC-specific, we performed k-means clustering of the differentially expressed genes (DEGs), separating patients with inflammatory conditions from healthy donors. This resulted in nine distinct modules based on different gene expression profiles across different sample groups (Figure 2A). These clusters are called modules in the remaining paper. They denote all genes with a similar gene expression profile but also imply resulting different protein-protein interaction networks participating each in a specific set of pathways (Figure 2B). Among these, four modules (1, 3, 7 and 8) were upregulated in TEPs from NSCLC compared to healthy samples and enriched in processes related to protein stability, platelet activation, metabolism, and cytoskeleton. In contrast, modules 2, 5, and 9 were downregulated and associated with processes related to ribosome, apoptosis, cell surface, DNA binding, metabolism, infection, and MHC class II complex (Figure 2B).

Module 1 was associated with nucleic acid and protein homeostasis as indicated by the overrepresented Gene Ontology Molecular Function terms (GO:MF) such as ATP-dependent chaperone, disaggregating activity, and DNA/RNA- and G-quadruplex-binding terms (Figure 2B). Among the genes-encoding proteins involved in these processes, we found drug targets like calcium voltage-gated channel subunit alpha1 D (*CACNA1D*), peroxisome proliferator activated receptor alpha (*PPARA*), lysophosphatidic acid receptor 4 (*LPAR4*), and malic enzyme 2 (*ME2*) (Table 1).

Genes in the high platelet activation-mediating module 3 were mostly upregulated in platelets from NSCLC patients and this module was the only module associated with many platelet-related REACTOME pathways like “Hemostasis”, “Platelet activation, signaling, and aggregation”, “Platelet degranulation”, and “Response to elevated platelet cytosolic Ca^2+^” (Figure 2B). In module 3, we found genes coding for monoamine oxidase B (*MAOB*), Fc gamma receptor IIa (*FCGR2A*), sphingomyelin phosphodiesterase 1 (*SMPD1*), glutathione S-transferase mu 3 (*GSTM3*), and integrin subunit alpha 2b (*ITGA2B*) as potential drug targets (Table 1). Apart from platelet pathways, the only other GO term overrepresented in this module was “Regulation of IGF and transport and uptake by IGFBPs”, and we found insulin-like growth factor-binding protein 2 (*IGFBP2*), *Laminin subunit beta-2* (*LAMB2*), interstitial collagenase (*MMP1*), and sulfhydryl oxidase 1 (*QSOX1*) being involved in this process (Table S2); however, no FDA-approved drugs were found targeting these proteins. This pathway also appeared among enriched Reactome pathways in cancer (*R-HSA-381426*, Datasheet S4).

Module 7 was predominantly associated with the metabolic adaptations in TEPs with overrepresentation of relevant KEGG and REACTOME pathways, as well as GO:MF and GO:CC terms (Figure 2B). We identified several druggable targets in this metabolic module, namely, carbonic anhydrase 1 (*CA1*), hemoglobin subunit alpha 1 (*HBA1*), SEC14 like lipid binding 2 (*SEC14L2*), and 5′-aminolevulinate synthase 2 (*ALAS2*) (Table 1).

Lastly, module 8 included genes involved in cytoskeletal remodeling of platelets, especially the spectrin and actin cytoskeleton (Figure 2B). This cytoskeletal module was enriched in GO terms associated with supramolecular fiber organization, cytoskeletal protein binding, and structural integrity of the platelet cytoskeleton. Among these genes, we identified antioxidant 1 copper chaperone (*ATOX1*), and microtubule-associated protein 1A (*MAP1A*) as potential drug targets (Table 1).

We identified four upregulated gene modules in NSCLC-derived TEPs: protein homeostasis (Module 1), platelet activation (Module 3), metabolism (Module 7), and cytoskeletal remodeling (Module 8). Each module contained potential drug targets, including *CACNA1D*, *FCGR2A*, *ITGA2B*, *CA1*, and *ATOX1*, highlighting key pathways for therapeutic exploration.

Building on the hierarchical clustering approach, we further examined the top upregulated REACTOME pathways (normalized enrichment score (NES) of at least 2, see Section 4), identified in gene set enrichment analysis (GSEA) to refine the selection of potential drug targets. Pathways related to extracellular matrix (ECM) remodeling, cell adhesion, and insulin signaling were among the most prominently enriched, indicating platelets’ contribution to cancer beyond their signaling. Given their functional relevance and overlap with KEGG and Gene Ontology categories, we prioritized druggable targets within these pathways (Table 2). Prevention of thrombosis, inflammation, as well as application of drugs targeting platelet hyperactivity, and at the same time the cancer cells directly (e.g., proliferation or metastasis targeting metalloproteases and cytoskeleton) are particularly beneficial for the patient.

Several proteins involved in ECM remodeling and adhesion were identified as potential therapeutic targets. Fibronectin (*FN1*) was highly represented in multiple pathways, including ECM proteoglycans and insulin-like growth factor (IGF) regulation, and is targetable by zinc-based compounds. Similarly, amyloid-beta precursor protein (*APP*) was enriched in ECM-associated processes and IGF transport, with known inhibitors such as deferoxamine and aducanumab. Plasminogen (*PLG*), which plays a role in ECM degradation and IGF transport, is targeted by fibrinolytic agents including, alteplase and urokinase among others. Basement membrane proteoglycan HSPG2 is another key component of ECM pathways and can be inhibited by palifermin and efanesoctocog alfa. Additionally, inter-alpha-trypsin inhibitor heavy chain H2 (ITIH2), present in IGF-related processes, is modulated by zinc acetate and zinc chloride.

Several additional pathway-linked targets were identified. Multiple sodium channel protein subunits (*SCN* genes) were significantly enriched in L1-ankyrin interactions and are known targets of brivaracetam, ranolazine, and cocaine. Proteins involved in cell junction organization, such as cadherin-11 (*CDH11*) and filamin A (*FLNA*), are targetable by celecoxib and artenimol, respectively. Moreover, collagen biosynthesis proteins (*P3H2*, *PLOD1*, *PLOD2*) were identified and can be modulated by ascorbic acid. Finally, coagulation factor V (*F5*), found in IGF regulatory pathways, is targetable by drotrecogin alfa and thrombin-based therapies (Datasheet S5).

These findings highlight key platelet-associated pathways in NSCLC and identify potential drug targets within ECM remodeling, adhesion, and insulin signaling.

In summary, these transcriptome-based findings provide an initial step in identifying potential therapeutic targets in NSCLC. While the clustering approach revealed platelet-intrinsic regulatory modules, GSEA uncovered globally enriched pathways in TEPs, representing broader systemic alterations in platelets in NSCLC. A more integrated perspective considering platelet interactions, pathway networks, and cancer cell targeting could further enhance the understanding and clinical relevance of these targets.

### 2.3. Therapeutic Target Discovery Based on Network Controllability

To have a broader view into platelet function in NSCLC, we first reconstructed the platelet signaling network using high-quality, directed, and signed interactions from OmniPath [76], which provides a comprehensive representation of key pathways involved in platelet function. This network consists of 962 interactions among 401 platelet-specific proteins, capturing essential signaling processes such as platelet activation, receptor tyrosine kinase signaling, and Rho GTPase signaling. Pathway enrichment analysis further highlights its strong representation of immune-related pathways and cytoskeletal regulation, reinforcing its biological relevance (Figure S2). The detailed construction and analysis of this network, along with its implications for platelet signaling, are presented in the Supplementary Text.

Our next step focused on analyzing the platelet network to determine its controllability, a key factor in understanding how to effectively manipulate the network, in other words, to be able to steer the network from any initial state to any desired final state through external interventions. To achieve this, we first identified critical nodes: nodes that are necessary for network control. These critical nodes are present in every MDS, meaning that they are necessary for modulating this network’s behavior. Our analysis revealed 86 critical nodes within the network. In addition, we identified 196 intermittent nodes that play a supporting role in network modulation and 119 redundant nodes, whose presence is not necessary for the network since alternative pathways exist.

**Figure 3:**
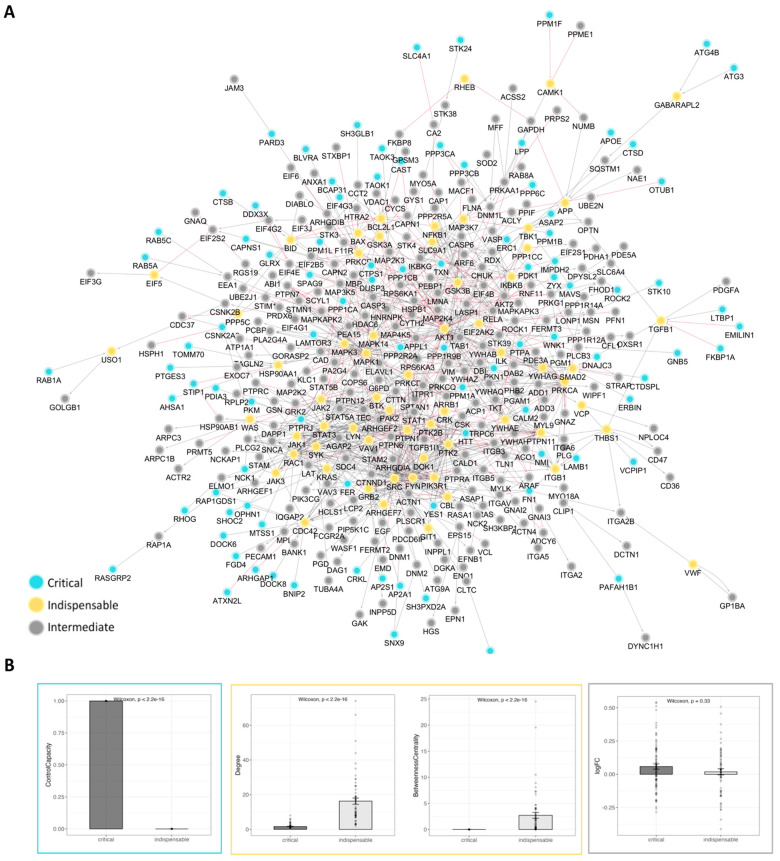
Network controllability analysis reveals key proteins to target in TEPs. (**A**) We show indispensable nodes (yellow) and critical nodes (blue) in the platelet network. (**B**) Comparison of controllability, degree, betweenness centrality, and logarithm of fold change (logFC) between indispensable and critical nodes. The statistical comparisons are made using the Mann–Whitney U test (Wilcoxon rank sum test).

Next, we analyzed the network to classify nodes according to their indispensability. Indispensable nodes are essential for signal transduction through the network; removing these nodes significantly hampers the ability to control the network. We found that the network contains 62 indispensable nodes. In contrast, we identified 127 neutral nodes, which have a moderate impact on control when removed, and 212 dispensable nodes, whose removal has little to no effect on the network’s overall controllability (Figure 3A).

To further understand the roles of critical and indispensable nodes within the platelet network, we compared their controllability and topological measures. Critical nodes are consistently included in the Minimum Steering Node Set (MSS), which is a subset of the Minimal Driver Sets (MDSs). Additionally, they show significantly higher control capacity. In contrast, indispensable nodes have a control capacity of zero and are not included in the MSS. On the other hand, indispensable nodes show a slightly higher average control centrality of 73.5 compared to the average control centrality of 69.3 for critical nodes (Figure 3B).

Topologically, the differences between these two types of nodes are also pronounced. Indispensable nodes have significantly higher values in various measures including degree (number of connections), closeness centrality, betweenness centrality, stress, and clustering coefficients. They also play a key role as partners in multi-edge node pairs. In contrast, critical nodes show higher eccentricity and average shortest path length (Figure S3). Critical nodes also have zero incoming interactions, and slightly lower neighborhood connectivity in comparison to indispensable nodes (Figure S4).

Interestingly, there is no significant difference between the average expression levels and log_2_FC values of critical and indispensable nodes (Figure 3B). This implies that the observed differences between these node types are not attributed to the inherent expression or differential expression of these nodes but rather stem from their distinct roles and functional positions within the network.

These findings underscore the distinct roles that critical and indispensable nodes play in the platelet network. While critical nodes are essential for network control, indispensable nodes contribute significantly to the network’s structural properties and connectivity.

**Figure 4:**
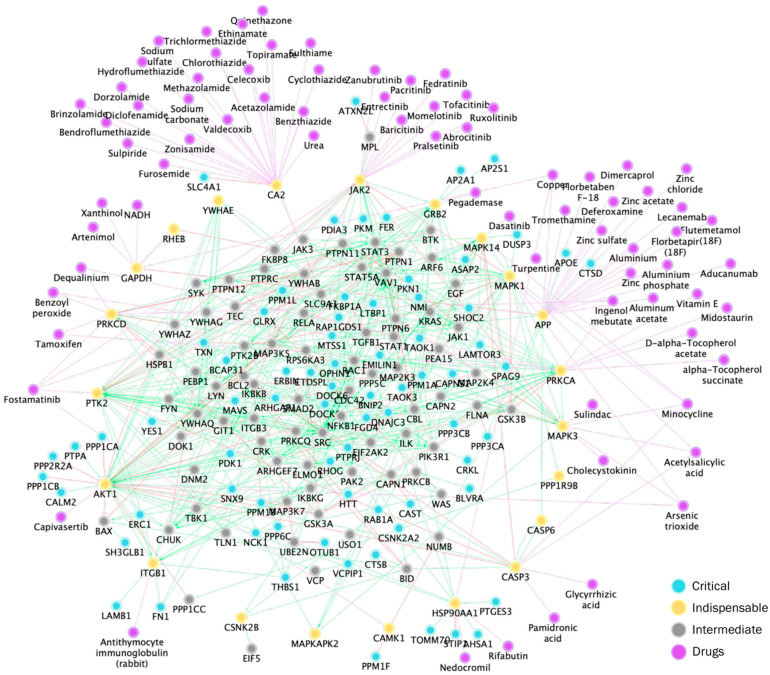
Central TEP subnetwork in the platelet signaling with key nodes and drugs to target them. The central TEP network was constructed by identifying shortest paths from critical to indispensable nodes targeted by FDA-approved drugs. Blue: critical, yellow: indispensable, gray: intermediate, purple: drugs.

To identify the most regulated and targetable regions of platelet signaling in NSCLC, we constructed a central TEP subnetwork by calculating edge weights based on the fold changes and mapping the shortest paths from critical to indispensable nodes. This resulted in a focused subnetwork of 188 nodes and 501 interactions (Figure 4), representing the key dysregulated platelet functions in the tumor environment.

To enhance specificity and safety of therapeutic options, we identified the indispensable nodes in this central TEP. With this approach, we aimed to narrow down the targetable space to those nodes that are more specifically involved in TEP functions, potentially offering a more precise and less risky therapeutic strategy.

Among the 22 indispensable nodes we identified in the central TEP subnetwork, 16 are known drug targets, which can be targeted by a total of 75 drugs. Top drugs targeting most indispensable nodes included fostamatinib, minocycline, and acetylsalicylic acid (Table 3). The top targets included were carbonic anhydrase 2 (*CA2*), amyloid-beta precursor protein (*APP*), and tyrosine-protein Janus kinase 2 (*JAK2*).

To prioritize clinically actionable therapies, we filtered for FDA-approved drugs and known pharmacological action to ensure clinical applicability. Of the 75 drugs initially identified, only 54 met this criterion. Among the indispensable nodes, there were no FDA-approved drugs with a known mechanism of action and a non-interacting profile for PRKCA, GAPDH, HSP90AA1, AKT1, GRB2, and ITGB1. We provide a list of drugs in the Supplementary Materials (Datasheet) that can target these nodes as their implied function in TEP signaling; these non-FDA-approved drugs are an important lead for later drug development, targeting in this way both TEP’s contribution metastasis, as well as direct metastatic potential in tumor cells.

To identify the most effective drug combination targeting indispensable nodes, we filtered for drugs with no interaction risk with fostamatinib. We then ranked combinations based on (1) number of indispensable targets covered, (2) target inhibition strength [79], and (3) control metrics of the targets within the network (e.g., control centrality, edge count, and betweenness centrality).

Fostamatinib emerged as the most promising agent, strongly inhibiting JAK2, PTK2, and CAMK1 (details in Supplemental Text). Aducanumab was selected for its specificity against APP, and Acetylsalicylic acid for targeting CASP3, both targets consistently ranking high in network influence.

While other drugs like minocycline and acetazolamide were considered, they either had weaker evidence or added to regimen complexity (details in Supplemental Text). By refining based on network metrics and drug interaction profiles, we propose the final, optimized combination of fostamatinib, aducanumab, and acetylsalicylic acid (aspirin) as a clinically actionable, multi-target strategy designed to modulate platelet hyperactivity and inflammation in cancer.

### 2.4. Expanded Platelet Interactome Reveals Novel Targetable Nodes

To enhance the platelet network analysis, we incorporated all available protein–protein interactions rather than restricting the dataset to signed and directed reactions from the Omnipath database. Filtering specific interactions can increase specificity but also carries the risk of overlooking potentially important correlations. By including all interactions, we reconstructed a more comprehensive platelet network encompassing 1638 interactions among 600 platelet proteins, approximately 1.5 times larger than the initial platelet signaling network.

To prioritize key proteins, we first assigned node weights based on both differential expression and network connectivity. The top 10% highest-weighted nodes (55 in total) included proto-oncogene tyrosine-protein kinase Src (*SRC*) as the most connected protein, followed by protein kinase C alpha (*PRKCA*), tyrosine-protein kinase Lyn (*LYN*), signal transducer and activator of transcription 1 (*STAT1*), and phosphoinositide-3-kinase regulatory subunit 1 (*PIK3R1*) (Table S3, Datasheet S7).

We then applied a second proximity-based scoring method designed for undirected networks, which ranked nodes by their closeness to high-weight nodes [80] (Table 4). The highest-scoring protein was phosphatidylinositol 3,4,5-trisphosphate 5-phosphatase 1 (known as *SHIP1* or *INPP5D*), followed by 1-phosphatidylinositol 4,5-bisphosphate phosphodiesterase gamma-2 (*PLCG2*) and E3 ubiquitin-protein ligase CBL (*CBL*), indicating their central roles in the expanded network (Table S4). This approach uncovered new potential drug targets, such as low-affinity immunoglobulin gamma Fc region receptor II-a (*FCGR2A*), P2Y purinoceptor 12 (*P2RY12*), tyrosine-protein kinase Tec (*TEC*), peptidyl-prolyl cis-trans isomerase A (*PPIA*), tyrosine-protein kinase CSK (*CSK*), phosphatidylinositol-4,5-bisphosphate 3-kinase catalytic subunit beta (*PIK3CB*), tubulin alpha-4A chain (*TUBA4A*), integrin alpha-5 (*ITGA5*), and tyrosine-protein phosphatase non-receptor type 6 (*PTPN6*) (Datasheet S8).

Among the high-score nodes, tyrosine-protein kinase JAK1 (*JAK1*) emerged as the most targetable, with ten FDA-approved drugs (according to DrugBank [81]) including ruxolitinib, tofacitinib, and baricitinib. Low-affinity immunoglobulin gamma Fc region receptor II-a (*FCGR2A*) was also highly targetable, with drugs such as cetuximab, bevacizumab, and etanercept. Similarly, tyrosine-protein kinase JAK3 (*JAK3*) was associated with multiple inhibitors, including ruxolitinib, filgotinib, and upadacitinib, while P2Y purinoceptor 12 (*P2RY12*) was targeted by ticagrelor, clopidogrel, and prasugrel. Notably, tyrosine-protein kinase BTK (*BTK*) was another promising target, with inhibitors such as ibrutinib, acalabrutinib, and zanubrutinib.

In conclusion, combining the results of new transcriptome data on tumor-educated platelets and combining it with latest proteome and protein interaction data, as well as applying a complementary prioritization approach revealed novel key nodes active in TEPs that are tumor -and metastasis-promoting, which, according to our results, are active in NSCLC lung cancer and we can now influence by FDA-approved drugs.

Several of the targetable genes identified are well-known players in normal hemostasis, such as *P2RY12 Lyn*, *Syk*, *Jak1*, *Jak3* and *FCGR2A*. Others are more involved in cytoskeletal signaling, such as *PIK3CB* and *BTK*. New targets suggested here involving more exclusively cytoskeletal signaling are *TUBA4A*, *ITGA5* and *PPIA*. Nevertheless, the network nodes are tightly connected and there is a certain overlap between all signaling networks in the platelet.

To validate the TEP targets from our study by independent new experimental datasets, we looked at the expression pattern for our reported genes in two other gene expression datasets, GSE183635 and GSE207586 (Datasheets S11 and S12). The log fold changes and, thus, the rankings among all the DEGs are almost the same for each of the found key genes *ITGA2B*, *FLNA*, *GRB2*, *FCGR2A*, and *APP* across all three datasets, confirming that these are consistently differentially expressed in NSCLC vs. healthy/non-cancer. As these are independent studies, findings here from these data show comparable results; this is a strong validation of our findings, in particular, regarding the expression of *ITGA2B*, *FLNA*, *GRB2*, *FCGR2A*, and *APP* as novel findings in these datasets.

In the transition to TEPs and to become cancer-promoting, first, proliferative acting transcription factors must be switched on. Several examples for transcription factors significantly upregulated in TEPs and not in normal platelets are apparent (see Supplementary Datasheet); for instance, *BTBD7P1* (BTB/POZ domain-containing protein 7; dataset DS1, line 60) which is upregulated 0.91 logarithm of fold change (logFC) in TEP compared to normal platelets. It acts as a mediator of epithelial dynamics and organ branching by promoting cleft progression and its expression in TEPs close to the tumor, if released (e.g., by activating the TEPs) is likely to promote tumor progression. Another example is *ZSCAN30* (see Dataset S1, line 16, zinc finger and SCAN domain-containing protein 30) upregulated 0.20 logFC in TEP compared to normal platelets.

Showing even more differences in TEPs compared to normal platelets, several zinc finger transcription factors such as *ZNRF2* (zinc and ring finger 2; Dataset S1, line 35) are downregulated. These TEP-specific transcription factors should already be active in megakaryocytes and stimulate a differentiation program different from normal platelet differentiation there.

The general tumor-educated platelet-targets and FDA-approved drugs targeting them and involved in hemostasis and cytoskeletal signaling are expected to hold for cancers in general, while the more NSCLC TEP-specific ones (e.g., *FLNA* and *APP*) could be more NSCLC-specific, for instance, not featuring in TEPs from pancreatic cancer [82].

To explore this, we looked at two further datasets (see Supplementary Datasheets S13 and S14):

- Datasheet S13 analyzes another TEP cohort (GSE68086; Best et al.) comparing breast cancer vs healthy. Results mirror our findings, with our top genes (ITGA2B, FLNA, GRB2, FCGR2A, APP) among the highest-ranking DEGs (|log_2_FC| ≈ 2–3).
- Datasheet S14 reuses the same GEO cohort (GSE183635) to compare pancreatic cancer with healthy controls. Our genes appeared again with very similar differential expression (often with slightly higher logFC), both across datasets and relative to each other.

The logFCs and placements among all the DEGs are only overall similar. These are shown in detail in Table 5 with the ranking of five key genes in the four datasets given, comparing two NSCLC datasets versus pancreatic and breast cancer. Though the ranking is of course not identical in the different datasets, the ranking of the genes in the three different datasets is broadly comparable among the top-ranked genes *ITGA2B*, *FLNA*, *GRB2*, *FCGR2A*, and *APP*. Except for a single outlying GRB2 value, the expression change is consistently significant and differs from controls across all four datasets, with effect sizes (fold changes vs. control) generally of similar magnitude. (see Table 5).

However, as there is clearly some variation among the datasets regarding the values and ranking, more data and further analyses are required to evaluate our top gene suggestions for targeting TEPs including data from different cancers, to allow further and more firm conclusions.

## 3. Discussion

Unlike physiological platelet activation triggered by vascular injury, tumor cell-induced platelet aggregation (TCIPA) is mediated by tumor-secreted factors such as coagulation and growth factors, and matrix metalloproteases [36,42]. This tumor-specific signaling presents a therapeutic opportunity to target tumor-educated platelets (TEPs) without disrupting normal platelet function [18].

Integration of the gene expression data with protein–protein interaction information allowed us to build a TEP-specific signaling network. Analyzing the network topology and controllability, we highlighted important proteins to this signaling, e.g., critical and indispensable nodes, high-weight and high-score nodes. These nodes revealed potential drug targets, and fostamatinib came up as the top-ranking with the broadest effect on the central TEP subnetwork.

The original authors (Best et al.) of the GSE dataset had an interesting agenda: “Swarm Intelligence-Enhanced Detection of Non-Small-Cell Lung Cancer Using Tumor-Educated Platelets”. Their study primarily analyzed RNA splicing events and identified differentially spliced transcripts in platelets from cancer patients using the thromboSeq method. They further focused on enhancing this approach with a particle swarm optimization (PSO) algorithm. However, their analysis was limited to splicing-related changes and did not include a detailed pathway analysis of platelet-pathophysiology. Instead, we directly investigated how TEP transcriptome differs from non-cancer platelets and activation.

Transcriptome comparisons revealed the highest similarity between TEPs and platelets from chronic pancreatitis patients, suggesting overlapping inflammatory signatures. However, to isolate cancer-specific signals, we pooled all non-cancer samples as controls. Among the most upregulated transcripts were several previously reported TEP biomarkers (e.g., *ITGA2B* (integrin alpha 2ß) [83], *CD79* (B-cell antigen receptor complex) [84,85], *PRSS50* (serine protease 50) [86], *CRYM* (crystallin mu; also known as THBP (NADP-regulated thyroid-hormone-binding protein)) [86], *IGFBP2* (insulin-like growth factor-binding protein 2) [86,87], *LGALS3BP* (galectin 3 binding protein) [87], *IFITM3* (interferon-induced transmembrane protein 3) [87], *HPSE* (heparanase) [87], *LAMB2* (laminin subunit beta 2) [86], *IFI27* (interferon alpha inducible protein 27) [87]). A notable novel finding was *PSG2* (Pregnancy-Specific Beta-1-Glycoprotein 2), a gene typically expressed in pregnancy [88] regulating immune response [89,90]. It was upregulated in certain cancers [91,92] but not previously detected in platelets. It has been noted that cancers exploit pregnancy-induced immunosuppression, which allows embryos and fetuses to express paternal antigens and still evade immune defense [93]. Its strong upregulation in TEPs, as well as lack of evidence to its presence in platelets, suggests tumor-derived RNA uptake, potentially supporting metastasis.

GSEA using Gene Ontology, Reactome Pathways, and KEGG databases revealed downregulation of RNA splicing and translation, as well as antigen processing and presentation, B- and T-cell receptor signaling, apoptosis, NF-κB signaling, antigen processing, apoptosis, TCA cycle, and oxidative phosphorylation, while we consistently observed upregulation of cell–cell or cell–ECM interaction, ECM organization, and cytoskeleton associated processes (Figure 1B). This finding aligns with decreased gene expression observed in lung cancer TEPs [15]. Additionally, Best et al. also identified cytoskeletal processes as upregulated, and RNA translation, T cell immunity, and interleukin signaling as downregulated in TEPs from NSCLC, as well as from glioblastoma, colorectal, pancreatic, hepatobiliary, and breast cancers [85].

Tumor-educated platelets (TEPs) showed transcriptional changes, indicating adhesive interactions, potentially with tumor cells and the vasculature, could promote in this combination metastasis. A key finding was the significant upregulation of *ITGA2B*, a subunit of the platelet-specific αIIbβ3 integrin complex. This integrin mediates platelet aggregation and binds to ECM proteins such as fibrinogen, von Willebrand factor (VWF), and fibronectin, all mildly upregulated in TEPs. These interactions facilitate tumor–platelet bridging via fibrinogen and αVβ3 on tumor cells [53,94,95], enhancing shear resistance and vascular adhesion representing key steps in extravasation [95,96]. Furthermore, we observed upregulation of other platelet surface proteins like glycoproteins, including *GP1BB* (glycoprotein Ib platelet subunit beta), *GPNMB* (glycoprotein nonmetastatic melanoma protein B), *GPM6A* (glycoprotein M6A), and *GPM6B* (glycoprotein M6B), as well as *PAPLN* (papilin; proteoglycan-like sulfated glycoprotein), *RHBG* (Rh family B glycoprotein), *SV2B* (synaptic vesicle glycoprotein 2B), *MOG* (myelin oligodendrocyte glycoprotein), and *PSG* (pregnancy-specific glycoprotein) *1*, *2*, *4*, *6*, *9*, and *11* (Figure S5).

TEPs showed increased expression of genes related to focal adhesion (KEGG hsa04510); proteins DOCK1 (dedicator of cytokinesis protein 1), LAMB2 (laminin subunit beta-2 protein), ITGA2B (integrin alpha 2ß), MYL9 (myosin light chain 9), FLNA (filamin A), and ECM–receptor interactions (map hsa04512; LAMB2, ITGA2B).

Conversely, key immune-related genes such as *CXCL8* (interleukin-8) were downregulated in TEPs. It is involved in neutrophil recruitment and angiogenesis [97,98,99], but its suppression may reflect tumor strategies to evade immune surveillance [100]. Gene Set Enrichment Analysis revealed consistent downregulation of RNA processing, immune signaling (T- and B-cell receptor pathways), and metabolism, while genes related to cytoskeletal remodeling and ECM interactions were upregulated—hallmarks of platelet activation and tumor interaction.

In our analysis of the platelet transcriptome in NSCLC, we observed downregulation of genes encoding several cell surface proteins in TEPs, contradicting increased cell adhesion processes. However, we mostly found downregulation of cell surface proteins related to immune signaling here. CD79B is a component of the B-cell antigen receptor complex, CD79, and plays a critical role in B-cell signaling. It has been identified as an oncogenic driver in lung adenocarcinoma [101] and is included among the eleven genes suggested by Best et al. [85] as biomarkers and then tested by Goswami et al. [84] for the TEPs to diagnose NSCLC. Both *CD79A* and *CD79B* are also part of the downregulated gene set of the KEGG pathway “B-cell receptor signaling” (Figure S6). We also found *CD8A* along with *CD8B*, *CD3D*, and *CD247* in the downregulated “T-cell receptor signaling” KEGG pathway in our enrichment analysis (Figure S6). Additionally, CXCR1 is a chemokine receptor, and the effect of platelets on T-cell CXCR1 has been shown to be immunosuppressive [102]. We observed CXCR1 in “Viral protein interaction with cytokine and cytokine receptor” KEGG pathway in GSEA (Figure S6).

In metastatic vs. primary cancer comparison (Datasheet S9), we found platelet-derived growth factor subunit A (*PDGFA*) upregulated (0.67). It was also slightly upregulated in NSCLC-TEPs when directly compared to healthy donors with no existing health condition (0.33) (Datasheet S10). PDGF is both pro-angiogenic and pro-metastatic [103] and increased PDGF secretion from platelets may be the distinguishing factor aiding the transition from primary tumor to metastatic tumor. Increased levels of PDGF, along with TGFβ and MMP1, was shown to result from platelets taking up mRNA and proteins that are secreted by tumors [22,23].

TEPs are enriched in IGF-related pathways (Figure S7), particularly the REACTOME pathway for “IGF transport and uptake via IGF binding proteins (IGFBPs)”, highlighting a potential role in cancer progression. IGF-1 enhances platelet activation through the IGF receptor/IRS/PI3K/PKB pathway [104], contributing to cancer-associated hypercoagulability [105] and metastasis [106]. Autocrine IGF-1 may further drive tumor-mediated platelet “education,” with cancer signals altering RNA splicing to increase IGF-related transcripts [106].

A key mediator is IGFBP2, upregulated in several cancers and involved in processes such as EMT, angiogenesis, and invasion via β-catenin and STAT2 signaling, contributing to the malignancy [107]. It promotes platelet-mediated cancer cell communication, particularly in MACC1-driven colorectal cancer metastasis [108,109]. IGFBP2 upregulation is further associated with increased metastasis, larger tumors, and poor survival in NSCLC [109,110,111], while enhancing gefitinib resistance through STAT2 signaling [112]. IGFBP2 can be released from platelets upon activation, leading to the modulation of IGF signaling in the tumor microenvironment [108] which suggests a role as a functional mediator as well as a biomarker for disease progression.

### 3.1. Targeting Tumor-Educated Platelet Signaling

TEPs have emerged as key facilitators of cancer progression, immune evasion, and metastasis. To identify potential therapeutic targets within TEPs, we employed four complementary strategies, integrating transcriptomic profiling with network-based systems biology approaches.

In the following, we show that our suggestions of targets and FDA-approved drugs to positively influence TEPs are supported by the literature.

The first two strategies focused exclusively on the gene expression data. These analyses identified *ITGA2B* and *FLNA* consistently upregulated and functionally relevant in platelet signaling and cancer-associated thrombosis. Both genes are critical to platelet structure and activation and represent targets of FDA-approved therapies.

Inhibitors of αIIbβ3 integrin were shown to decrease TCIPA and ECM degradation by tumor cells [94], and colonization of tumor cells in the lungs [70]. Moreover, the strong expression levels of *ITGA2B* were found to be significantly higher in TEPs from NSCLC patients, and its use as a diagnostic marker proved to have high sensitivity and specificity, making it a promising marker to diagnose early-stage NSCLC [83].

To expand beyond expression changes and capture the complexity of protein interactions, we implemented two network-based approaches. In the third strategy, we applied network controllability analysis, identifying optimal intervention points that could efficiently modulate TEP signaling. This approach prioritized a drug combination of fostamatinib, aducanumab, and acetylsalicylic acid, targeting *JAK2*, *PTK2*, and *CAMK1*; *APP*; and *CASP3*, respectively.

The fourth strategy involved a network proximity-based scoring method to rank genes by their closeness to high-weight nodes within the platelet–cancer interaction network. Both network-based strategies independently highlighted GRB2, a central adaptor in multiple platelet activation pathways, including ITAM signaling, as a potential therapeutic bottleneck [113,114].

Across all methods, we observed convergence on FCGR2A and APP as key targets, supporting their robust involvement in cancer–platelet signaling. APP, known for its role in amyloid-β processing, also contributes to platelet-mediated thrombus formation and is targetable via monoclonal antibodies such as aducanumab [115].

FCGR2A can be engaged by tumor cell-derived IgG/immune complexes to facilitate platelet activation via ITAM signaling [35]. Importantly, FcγRIIA and αIIbβ3 (ITGA2B) are canonical platelet activation routes and in this context, FcγRIIA signals through ITAM adaptors, whereas αIIbβ3 mediates outside-in integrin signaling; GRB2 functions as a shared adaptor across multiple pathways [116]. FCGR2A-dependent activation has been implicated to mediate immuno-thrombosis across multiple cancers via interacting with the platelet integrin αIIbβ3, though not yet in NSCLC [35,116]. Studies have shown that blocking FCGR2A or knocking down IgG in various cancers, including hepatocellular carcinoma (HCC), cervical, and bladder cancers, reduces platelet activation and subsequent metastasis [35]. Similarly, in colon, prostate, and breast cancers, interference with FCGR2A has proven effective in limiting platelet activation [34]. However, the role of FCGR2A in NSCLC remains unexplored. Targeting cancer-derived IgG or blocking FCGR2A could potentially hinder TEP-mediated metastasis and may offer a novel therapeutic strategy for inhibiting cancer-induced platelet activation while minimizing disruption to normal hemostatic processes.

Taken together, our integrative strategy revealed ITGA2B, FLNA, GRB2, FCGR2A, and APP as high-confidence, FDA-targetable candidates that intersect cancer-associated platelet education with pro-metastatic platelet signaling.

The ranking of the five genes, is of course, not identical in the four different datasets compared in Table 5; however, the overall values, effect size (log FC) and placement among top-ranked genes are generally the same (with one value for *GRB2* being an exception). Given the typical error from such gene expression measurements, it is completely sufficient to pick these specific genes as potentially strong mediators of TEP effects for further investigation in other datasets and targeted experiments testing gene effects (e.g., by knocking one of the five genes out).

Importantly, several targets (e.g., GRB2, FCGR2A) are involved in ITAM signaling, which governs immune receptor-driven platelet activation. Selectively disrupting this pathway may suppress TEP-mediated cancer progression while preserving physiological hemostasis.

Along with the targeted therapeutic strategies focusing on specific platelet proteins, most of which proved to be useful targets in anti-platelet and anti-metastatic therapy [41,63,64,65,66,67,68,69,70,71,72,73,74,117,118,119,120], we looked at the bigger picture using a more comprehensive strategy by investigating the TEP signaling regulated by cancer-employing networks. Across network-based strategies, fostamatinib frequently emerged as a top drug target, as well as in our previous study on platelet hyperactivation in COVID-19 [77]. Although we identified multiple targets of fostamatinib in our networks, it is important to note that it does not inhibit all targets equally, as indicated by varying inhibition constants (K_i_) [79]. Through its primary target Syk, it inhibits the ITAM signaling pathways downstream of GPVI, CLEC-2, and FCGR2A [121,122,123]. These three receptors were also shown to be the primary ways tumor cells activate platelets, different from physiological conditions, where platelet activation starts with adhesion receptors [32,33,34,124]. Note: these receptors are not tumor-specific modes of engagement—they are used by other activators as well. Furthermore, it has been reported that both liver cancer [35] and SARS-CoV-2 [125,126] activate platelets by IgG binding to the FCGR2A receptor. This suggests that receptors of ITAM signaling are promising drug targets for managing both infection-related coagulopathy and cancer-associated thrombosis. In particular, inhibiting GPVI was shown to inhibit thrombosis with no major impact on hemostasis, making it a potential anti-thrombotic and anti-metastatic therapeutic option [32].

Furthermore, we suggest a combination therapy of fostamatinib, aducanumab, and aspirin, tailored to selectively target TEP activity instead of only using fostamatinib. As they do not interfere with each other and act independently with different biological targets (see Supplementary Text), this allows a safer low-dose regime for administering the combination.

Fostamatinib has already been shown to be a safe and effective therapeutic agent against primary and secondary immune thrombocytopenia (ITP) [127]. Besides the anti-thrombotic features, it demonstrated anti-cancer activities in multiple in vitro studies, inhibiting various molecules and pathways such as receptor tyrosine kinases, PI3K-AKT pathway, and immune checkpoints PD-L1 and CD47, decreasing cell proliferation and inducing apoptosis in different cancers, including NSCLC and AML [128,129]. These results were also supported by multiple phase I and phase II clinical trials targeting different carcinomas such as NSCLC, advanced colorectal, platinum-resistant ovarian, thyroid, and renal cell carcinoma [78,130].

Aspirin has long been known to show anti-platelet activity mainly via the inhibition of COX-1 pathway and reducing thromboxane A2 formation, and thus, has been effective against inflammation-induced platelet aggregation [131,132,133]. It was also suggested to have a protective effect against multiple cancer types, including colorectal, prostate, and pancreatic cancers as well as lymphomas [134]. A recent study revealed anti-metastatic properties of aspirin via the inhibition of COX-1-TXA2 pathway and releasing T-cells from the TXA2-mediated and intrinsic ARHGEF1-dependent immunosuppression, leading to decrease in metastatic rates [135]. When used in combination, the anti-thrombotic and anti-metastatic properties of fostamatinib and aspirin might be boosted as the safety of such a combination was previously assessed positively [122]. Aducanumab has not yet been tested for any effect regarding platelets or cancer; however, it might be a valuable addition against one of the consistently emerging key proteins, APP, that aducanumab was shown to effectively target [136].

Based on these observations, the potential of fostamatinib against platelet hyperactivation in inflammatory contexts is strongly supported. As the next steps, experiments to assess the effects of fostamatinib on the other targets that we identified can help refine our networks and followingly our understanding of their impact. Additionally, measuring fostamatinib’s impact directly on platelet function in platelets derived from NSCLC patients would further reinforce its anti-metastatic use, while in the context of COVID-19, it has already been shown to reverse platelet hyperactivity [123]. It is important to note that using drugs with broader effects on TEP signaling may have drawbacks, including the possibility of unknown side effects that could lead to bleeding complications. Therefore, future work should focus on functional validation of these targets, particularly in NSCLC, and assessment of therapeutic combinations in preclinical models.

### 3.2. Limitations and Outlook

To gain a broader understanding of the cancer side of the platelet–cancer loop, reconstruction of the NSCLC large-scale networks can help identify the significant modules when combined with gene expression or proteomics data. Additionally, as we demonstrated in the analysis of the platelet signaling, network analysis including centrality and controllability measures can assist in identifying key targets that are central to pro-metastatic functions. Our data suggest that the results are robust in different NSCLC datasets and the key target genes identified (ITGA2B, FLNA, GRB2, FCGR2A, and APP, etc.) seem to also be strongly expressed in the breast cancer and pancreatic cancer datasets we analyzed. This is quite promising but needs more detailed investigation to be sure.

Further investigation of platelets’ role in metastasis requires drawing a more specific picture. For this, although more challenging than large-scale analysis, integration of newly found important platelets protein and their simulation may be helpful. Furthermore, a complete annotation of platelet proteins such as receptor, surface, or secreted proteins, etc., can help to identify important surface proteins. These can be then investigated for their interaction partners on cancer cell surface via analyzing domain–domain interactions, domain–motif interactions, and using machine learning approaches.

Lastly, investigating the indirect role of platelets in cancer through interactions with other cell types may help us understand their functions in a bigger context, including interactions with endothelial cells to promote angiogenesis or natural killer cells to inhibit their cytotoxicity or macrophages to induce their differentiation to M2 phenotype, all of which constitute an important aspect of platelets’ role in metastasis.

Mammalian platelets evolved to be highly efficient in hemostasis and immune defense, offering survival advantages through rapid response to injury; however, this efficiency carries an evolutionary tradeoff, which is particularly evident under chronic inflammatory conditions that now drive high rates of thrombotic disorders in humans [137]. With advances in medicine significantly increasing life expectancy since the 19th century [138,139], infectious diseases have become less prevalent, shifting major health burdens toward aging-related conditions like cardiovascular disease and cancer [140]. As a result, platelet hyperactivity has emerged as a central factor in these diseases, as well as in acute infections such as COVID-19 [141,142,143]. The very mechanisms that once ensured survival are now co-opted by cancers, which mimic chronic wounds [144] and perpetuate a cycle of inflammation and platelet activation [145], fostering disease progression through thrombo-inflammation. Thus, our drug screening approach, while directly targeting TEPs, also potentially disrupts pro-metastatic tumor cell behaviors and coagulation-promoting kinase activity, stressing a dual therapeutic benefit.

## 4. Materials and Methods


**Overview on used software and Databases**

**Softwares and algorithms**

**Ref.**

**Source Identifier**
conda 24.3.0[146]
https://docs.anaconda.com/
cowplot 1.1.3[147]
https://CRAN.R-project.org/package=cowplot
CytoCtrlAnalyzer[148]
https://apps.cytoscape.org/apps/cytoctrlanalyser
Cytoscape 3.10.2[149]
https://cytoscape.org/
Cytoscape Omnipath[150]
https://apps.cytoscape.org/apps/omnipath
DEFormats 1.30.0[151]
https://bioconductor.org/packages/release/bioc/html/DEFormats.html
DESeq2 1.42.1[152]
https://bioconductor.org/packages/release/bioc/html/DESeq2.html
edgeR 4.0.16[153]
https://bioconductor.org/packages/release/bioc/html/edgeR.html
Enhancedvolcano 1.20.0[154]
https://bioconductor.org/packages/release/bioc/html/EnhancedVolcano.html
EnsDb.Hsapiens.v86 2.99.0[155]
https://bioconductor.org/packages/release/data/annotation/html/EnsDb.Hsapiens.v86.html
fastp 0.23.4[156]
https://anaconda.org/bioconda/fastp
FastQC 0.12.1[157]
https://anaconda.org/bioconda/fastqc
ggplot2 3.5.1[158]
https://CRAN.R-project.org/package=ggplot2
ggpubr 0.6.0[159]
https://CRAN.R-project.org/package=ggpubr
ggraph 2.2.1[160]
https://CRAN.R-project.org/package=ggraph
glmGamPoi 1.14.3[161]
https://bioconductor.org/packages/release/bioc/html/glmGamPoi.html
gprofiler2 0.2.3[162]
https://CRAN.R-project.org/package=gprofiler2
gt 0.11.0[163]
https://CRAN.R-project.org/package=gt
igraph 2.0.3[164]
https://CRAN.R-project.org/package=igraph
kallisto 0.50.1[165]
https://anaconda.org/bioconda/kallisto
limma 3.58.1[166]
https://bioconductor.org/packages/release/bioc/html/limma.html
MetBrewer 0.2.0[167]
https://CRAN.R-project.org/package=MetBrewer
MultiQC 1.22.2[168]
https://anaconda.org/bioconda/multiqc
OmniPathR 3.10.1[169]
https://bioconductor.org/packages/release/bioc/html/OmnipathR.html
org.Hs.eg.db 3.18.0[170]
https://bioconductor.org/packages/release/data/annotation/html/org.Hs.eg.db.html
pheatmap 1.0.12[171]
https://CRAN.R-project.org/package=pheatmap
plotly 4.10.4[172]
https://github.com/plotly/plotly.R
R 4.3.0 (2023-04-21)—“Already Tomorrow”N/A
https://cran.r-project.org/
rhdf5 2.46.1[173]
https://www.bioconductor.org/packages/release/bioc/html/rhdf5.html
RStudio 2024.04.2+764 (2024.04.2+764)[174]
https://posit.co/download/rstudio-desktop/
RUVSeq 1.36.0[175]
https://bioconductor.org/packages/release/bioc/html/RUVSeq.html
SANTA 2.38.0[80]
https://bioconductor.org/packages/release/bioc/html/SANTA.html
tidyverse 2.0.0[176]
https://cran.r-project.org/web/packages/tidyverse/index.html
tximport 1.30.0[177]
https://bioconductor.org/packages/release/bioc/html/tximport.html
swamp 1.5.1[178]
https://CRAN.R-project.org/package=swamp

**Databases**

Drugbank 5.0[81]
https://go.drugbank.com/
Scripts for all the analyses can be found at https://github.com/ozgeosmanoglu/Platelet_NSCLC. All source identifiers verified on 28 October 2025.

### 4.1. RNAseq Data Analysis

We downloaded 779 samples (826 SRA files/runs) from ENA Bioproject ID: PRJNA353588, GSE89843 [75]. The samples included 402 tumor-educated platelet (TEP) samples from patients with non-small-cell lung cancer (NSCLC) and 377 platelet samples from donors without cancer, some with inflammatory conditions. The samples were sequenced on Illumina Hiseq 2500 platform. The quality control of the fastq files was evaluated with FastQC [157] and fastp [156]. Fastp was also used to trim adapters and low-quality reads. After trimming, we indexed human GRCh38 cDNA/transcriptome with kallisto index and performed pseudoalignment and quantification of the reads with kallisto quant function [165].

### 4.2. Differential Gene Expression Analysis and Data Normalization

Kallisto output files with counts for each sample were imported in Rstudio with tximport [177]. We first collapsed technical replicates (*collapseReplicates* from DESeq2) and removed one sample from PISA hospital (GSM2391029). Lowly expressed genes were filtered by using *filterByExpr* from edgeR (min.count = 10, design = ~class, min. group size = 376). We performed variance-stabilizing transformation (vst from DESeq2) to explore the data with principal component analysis (PCA). We removed two outliers we observed in PCA plots (GSM2390915 and GSM2627443). Next, we checked for unwanted variation (UV) in the data using *RUVg* from RUVSeq. For this, we generated a list of stable (empirical) genes. We fit a Gamma–Poisson generalized linear model to the filtered data and ran a likelihood ratio test (LRT) to find genes that do not have changes in their expression across different levels of the experimental conditions (~class). We then defined the empirical genes as the genes with log_2_FC between −0.05 and 0.05 and with adjusted *p*-values larger than 0.05. We ended up with 1491 genes as empirical genes and using this list, we performed RUV with k = 5. We included the 5 UV factors in the design matrix alongside the condition of interest (~class: non-cancer vs. NSCLC) and performed differential expression (DE) analysis with limma-voom and edgeR. Genes that have log_2_FC values smaller than −0.58 and larger than 0.58 adjusted *p*-values smaller than 0.05 were classified as differentially expressed genes.

### 4.3. Volcano Plots and Heatmaps

We used EnhancedVolcano R package version 1.20.0 to make the volcano plots of all the genes. Differentially expressed genes were colored blue if downregulated, red if upregulated, black if significant, and gray if not significant.

For the heatmaps, we used pheatmap and scaling was performed row-wise (to obtain z-scores).

### 4.4. Gene Set Enrichment Analysis

We used ClusterProfiler version 4.10.1 for the Gene Set Enrichment Analysis (GSEA) and used enrichGO, enrichKEGG functions for Gene Ontology and KEGG Pathways. For Reactome Pathways, the gsePathway function from ReactomePA R package version 1.54.0 was used. *p*-values were corrected for multiple testing using Benjamini–Hochberg.

### 4.5. Clustering of DEGs

We used k-means clustering with k = 9 (number of modules) to cluster DEGs. We separated the non-cancer class into subgroups and ended up with 9 groups: NSCLC, Healthy, Chronic Pancreatitis, Epilepsy, Multiple Sclerosis (MS), Non-significant (NS) atherosclerosis, Pulmonary Hypertension, Stable Angina Pectoris (AP), and Unstable Angina Pectoris (AP). We used Pearson correlation to cluster the rows and Spearman correlation for samples. For each module, we then performed functional enrichment analysis using the *gost* function from gprofiler2 package to find overrepresented Gene Ontology (GO) terms, KEGG, and REACTOME pathways. We visualized the top 5 overrepresented pathways for each module using ggplot2.

### 4.6. Construction of Platelet Signaling Network

We began by importing all post-translational interactions using the OmnipathR version 3.10.1 [169] package in R. To ensure data quality, we filtered out low-quality interactions and kept only directed and signed interactions that were supported by a curation effort of at least 3 [76]. This quality control step significantly reduced the number of interactions from 134,282 to 12,963.

Next, we constructed the platelet signaling network by filtering the high-quality Omnipath interactions based on gene and protein expression specific to platelets. We selected nodes with an average expression (average log_2_fold expression for the probe over all arrays and channels in limma:topTable) of at least 1 (25th percentile, also defined as first quartile) in the RNA-sequencing dataset or those detected in the two proteomics datasets (unpublished, details in Supplement Materials). We then predicted interactions based on the regulation of the nodes involved by multiplying their respective log_2_FC values. We used only the log_2_FCs of significant genes, as the regulation of non-significant genes could be misleading; for these, we assigned NA. If the product of this multiplication was positive, the interaction was classified as “activation”; if negative, it was classified as “inhibition.” We then compared these predicted interactions to the actual interaction types from the Omnipath database. Interactions where the predicted and actual types were contradictory were removed from the network, and the nodes that were not connected with the rest of the network were removed, resulting in the largest connected component of the platelet network containing 962 interactions among 401 platelet proteins.

From this platelet network, we further extracted a subnetwork focused on differentially expressed genes (DEGs). We isolated the interactions involving the 219 DEGs and removed any interactions that contradicted the predicted interaction type, as previously described. This process yielded a DEG network consisting of 23 nodes and 31 interactions.

Pathway Overrepresentation Analysis was performed using gProfiler Cytoscape plugin. *p*-values were adjusted using g:SCS algorithm and whole human genome was used as background.

### 4.7. Network Controllability and Subnetworks

We identified the nodes that are topologically important for the controllability of the platelet network: indispensable nodes, and critical nodes.

To identify the minimum driver node sets (MDSs) in the reconstructed networks, we implemented the concept of controllability and used Maximum Matching (MM), a graph theory-based approach for network analysis [148]. Then, using the identified MDSs, we utilized the node classification schemes recommended by Vinayagam et al. [179], i.e., indispensable, neutral, and dispensable, and Jia et al. [180], i.e., critical, intermittent, and redundant, to identify the key nodes in the network for controllability and targeting.

To obtain the controllable subnetworks, we first calculated edge weight between nodes *i* and *j* as follows:

(1) $$W_{ij}=\frac{1}{\left|{log}_{2}{FC}_{i}*{log}_{2}{FC}_{j}\right|}$$

A lower edge weight signifies a more important edge, where the interacting nodes exhibit higher relative fold changes between two conditions. Using these edge weights, we identified the shortest paths from critical nodes to indispensable nodes by applying Dijkstra’s algorithm (igraph::shortest_paths) and constructed the controllable subspace.

### 4.8. Gene Scores and Subnetworks

We first assigned node weights to each node in the platelet network using the multiplication of degree and log_2_FC. For nodes that were not significantly regulated, we used log_2_FC value of 1 × 10^−6^. High-weight genes are the ones that are then highly connected and regulated. Then, using these high-weight genes, we assigned gene scores to nodes that are connected to them with shortest path.

(2) $$K_{i}^{node}\left[s\right]=\frac{2}{p}\;\sum_{j}\;(p_{j}-\underline{p})I(d^{g}\;(i,j)\leq s)$$

where $p_{j}$ represents the weight of node j and $\underline{p}$ is the average node weight, and $I(d^{g}\;(i,j)\leq s)$ is an identity function that equals 1 when node I and node j are within a distance s, and 0 otherwise (SANTA::Knode) [80]. We then obtained controllable subspaces by combining the shortest paths between the top 10% of the high-gene-scored nodes and the top 10% high-weight nodes. Since the node classification is already based on differential regulation, we did not use edge weights.

### 4.9. Drug Repurposing

We downloaded all FDA-approved drugs and their targets from Drugbank 5.1.12. We searched for all drugs that target the critical nodes (or the high-gene-score nodes) in the controllable subspace and visualized it with R ggraph. Drug interactions were obtained from Drugbank and drugs.com.

## 5. Conclusions

Tumor-educated platelets (TEPs) display a cancer-specific transcriptional program distinct from physiological activation. In this study, we investigated TEPs in non-small-cell lung cancer (NSCLC) to uncover potential therapeutic vulnerabilities that could curb metastasis without impairing physiological hemostasis. Analyzing an exceptional, recent large-scale transcriptome dataset focusing on TEPs, and complementing it with further recent transcriptome data-sets and the latest data on protein–protein interactions, we give pathways, molecular markers, and target TEPs pharmacologically in a novel, beneficial way which, after appropriate clinical testing, should find its way into the clinical setting.

**Pathways:** Transcriptomic analysis of latest TEP data revealed that genes for adhesion, ECM-receptor interactions, and cytoskeletal remodeling are upregulated, whereas RNA processing, antigen presentation, lymphocyte receptor signaling, and core metabolic pathways are suppressed.

**Molecular markers:** Alongside established TEP biomarkers (ITGA2B, IGFBP2), PSG2 indicates tumor-derived RNA uptake. PDGF/IGF signaling may potentiate platelet activation and pro-metastatic crosstalk. In this platelet state primed for tumor binding, vascular adhesion, and metastatic assistance, central platelet subnetworks involve the ITAM-SYK hyperactivation core (FCGR2A, SYK, GRB2) with JAK2, CAMK1, and PTK2 as potential intervention points; a cytoskeleton/adhesion sub-module (ITGA2B, FLNA, MYL9, and DOCK1), an ECM-remodeling/metalloprotease axis (MMP1, HPSE), and an apoptosis sub-module centered around CASP3. Two approaches based solely on differential gene expression converged on ITGA2B and FLNA, known regulators of platelet structure and function. Network-based analyses, leveraging controllability and proximity scoring, further highlighted GRB2 as a key signaling hub. Importantly, FCGR2A and APP were consistently identified across transcriptomic and network methods, reinforcing their roles in TEP-mediated immune evasion and thrombo-inflammatory processes.

**FDA-approved drugs for mitigation:** These five genes—*ITGA2B*, *FLNA*, *GRB2*, *FCGR2A*, and *APP*—emerged as high-confidence, FDA-targetable candidates. Many participate in ITAM signaling, which is uniquely co-opted by tumor cells to activate platelets, representing an attractive avenue for targeted disruption of cancer-associated platelet activity. We also identify metalloproteases as key promoters of metastasis and historically challenging, yet compelling targets for therapeutic exploration. Notably, fostamatinib, an FDA-approved SYK inhibitor, consistently ranked as a top candidate, suggesting it could suppress ITAM-mediated TEP activation. In light of its previous success in reversing platelet hyperactivation in COVID-19, we propose fostamatinib, alone or in combination with aducanumab and acetylsalicylic acid, as a potential therapeutic strategy to selectively block cancer–platelet crosstalk. Future experimental validation in NSCLC-derived platelets and preclinical models will be critical to assess the efficacy and safety of these targeted interventions and to refine our understanding of TEP signaling in cancer progression. 

## Figures and Tables

**Table 1:** Drug targets in the upregulated modules in TEPs.

Target Name ^1^	Description	Drugs ^2^	Module
*CACNA1D*	calcium voltage-gated channel subunit alpha1 D	Ergocalciferol, Enflurane, Ranolazine, Phenytoin, Isradipine, Topiramate, Nimodipine, Nisoldipine, Spironolactone, Nicardipine, Magnesium sulfate, Verapamil, Levomenthol, Ethanol, Felodipine, Miconazole, Nifedipine, Amiodarone, Dronedarone, Clevidipine, Levamlodipine, Fish oil	1
*PPARA*	peroxisome proliferator-activated receptor alpha	Valproic acid, Indomethacin, Rosiglitazone, Fenoprofen, Fenofibrate, Ibuprofen, Amiodarone, Gemfibrozil, Prasterone, Palmitic Acid, Soybean oil, Fenofibric acid, Fish oil	1
*LPAR4*	lysophosphatidic acid receptor 4	Promethazine	1
*ME2*	malic enzyme 2	NADH	1
*MAOB*	monoamine oxidase B	Amphetamine, Phentermine, Procaine, Tranylcypromine, Phenelzine, Zonisamide, Selegiline, Pioglitazone, Procarbazine, Isocarboxazid, Rasagiline, Metamfetamine, Flavin adenine dinucleotide, Safinamide, Viloxazine, Flortaucipir F-18	3
*FCGR2A*	Fc gamma receptor IIa	Cetuximab, Etanercept, Human immunoglobulin G, Abciximab, Alemtuzumab, Bevacizumab, Sarilumab	3
*SMPD1*	sphingomyelin phosphodiesterase 1	Amlodipine, Chlorpromazine, Desipramine	3
*GSTM3*	glutathione S-transferase mu 3	Glutathione disulfide, Deoxycholic acid	3
*ITGA2B*	integrin subunit alpha 2b	Abciximab, Tirofiban	3
*CA1*	carbonic anhydrase 1	Topiramate, Chlorthalidone, Amlodipine, Methocarbamol, Bendroflumethiazide, Methazolamide, Hydroflumethiazide, Acetazolamide, Dorzolamide, Chlorothiazide, Zonisamide, Diclofenamide, Brinzolamide, Sodium sulfate	7
*HBA1*	hemoglobin subunit alpha 1	Iron Dextran, Nitrous acid, Copper, Sodium ferric gluconate complex, Ferric pyrophosphate citrate, Zinc acetate, Ferrous fumarate, Zinc chloride, Voxelotor, Ferric derisomaltose	7
*SEC14L2*	SEC14-like lipid binding 2	Vitamin E	7
*ALAS2*	5′-aminolevulinate synthase 2	Glycine	7
*ATOX1*	antioxidant 1 copper chaperone	Cisplatin	8
*MAP1A*	Microtubule-associated protein 1A	Estramustine	8

^1^ FDA-approved drugs from Drugbank v5.1.12. ^2^ Only upregulated module genes are targeted.

**Table 2:** Drug targets in the upregulated REACTOME pathways in TEPs.

ID	Description	Drug Targets
R-HSA-445095	Interaction between L1 and ankyrins	*SCN1A* (sodium channel protein type 1 subunit alpha), *SCN2A* (sodium channel protein type 2 subunit alpha), *SCN9A* (sodium channel protein type 9 subunit alpha), *SCN3A* (sodium channel protein type 3 subunit alpha), *SCN11A* (sodium channel protein type 11 subunit alpha), *SCN8A* (sodium channel protein type 8 subunit alpha), *SCN1B* (sodium channel regulatory subunit beta-1), *SCN3B* (sodium channel regulatory subunit beta-3)
R-HSA-3000178	ECM proteoglycans	*APP* (amyloid-beta precursor protein), *FN1* (fibronectin), *HAPLN1* (hyaluronan and proteoglycan link protein 1), *HSPG2* (basement membrane-specific heparan sulfate proteoglycan core protein), *ITGA2B* (integrin alpha-IIb)
R-HAS-1474228	Degradation of the extracellular matrix	*ELN* (elastin), *FBN2* (fibrillin-2), *FN1* (fibronectin), *HSPG2* (basement membrane-specific heparan sulfate proteoglycan core protein), *NID1* (nidogen-1), *PLG* (plasminogen)
R-HSA-446728	Cell junction organization	*CDH11* (cadherin-11), *FLNA* (filamin-A), *TESK1* (dual specificity testis-specific protein kinase 1)
R-HSA-381426	Regulation of fIGF transport and uptake by IGFBPs	*APP* (amyloid-beta precursor protein), *CP* (ceruloplasmin), *F5* (coagulation factor V), *FN1* (fibronectin), *ITIH2* (inter-alpha-trypsin inhibitor heavy chain H2), *PLG* (plasminogen), *SERPIND1* (heparin cofactor 2)
R-HSA-5173105	O-linked glycosylation	*MUC16* (mucin-16)
R-HSA-1474290	Collagen formation	*P3H2* (prolyl 3-hydroxylase 2), *P4HB* (protein disulfide-isomerase), *PLOD1* (procollagen-lysine,2-oxoglutarate 5-dioxygenase 1), *PLOD2* (procollagen-lysine,2-oxoglutarate 5-dioxygenase 2)
R-HSA-1592389	Activation of matrix metalloproteinases	no FDA-approved drugs found.

**Table 3:** FDA-approved drugs targeting the reanalyzed indispensable nodes in the controllable Subnetwork.

Drug Name ^1^	Number of Targets	Targets ^2^
Fostamatinib	5	CAMK1, JAK2, MAPK14, PRKCD, PTK2
Minocycline	4	CASP3, MAPK1, MAPK14, MAPK3
Acetylsalicylic acid	3	CASP3, MAPK1, MAPK3
Arsenic trioxide	3	MAPK3, MAPK1, AKT1
Copper	3	APP, GAPDH, HSP90AA1
Benzoyl peroxide	2	PRKCA, PRKCD
Dequalinium *	2	PRKCA, PRKCD
Ingenol mebutate	2	PRKCD, PRKCA
Tamoxifen	2	PRKCA, PRKCD
Abrocitinib	1	JAK2

^1^ FDA-approved drugs from Drugbank v5.1.12. ^2^ Only reanalyzed indispensable nodes are targeted. * Other-approved.

**Table 4:** Targetable high-score nodes in platelet interactome.

Drug Name ^1^	Degree	Log_2_FC	Weight	Gene Score	Drugs ^2^
LYN	48	−0.24	11.34	0.48	Dasatinib, Bosutinib, Ponatinib, Nintedanib, Fostamatinib
JAK1	21	--	0	0.47	Ruxolitinib, Tofacitinib, Momelotinib, Baricitinib, Fostamatinib, Fedratinib, Filgotinib, Abrocitinib, Upadacitinib, Pralsetinib
FCGR2A	4	0.68	2.71	0.46	Cetuximab, Etanercept, Human immunoglobulin G, Abciximab, Alemtuzumab, Bevacizumab, Catumaxomab, Sarilumab
TEC	10	0.25	2.47	0.46	Bosutinib, Fostamatinib, Ritlecitinib, Zanubrutinib
TUBA4A	1	−0.13	0.13	0.45	Vincristine, Podofilox
PTPN6	29	−0.09	2.73	0.45	Tiludronic acid
SYK	37	−0.1	3.84	0.45	Fostamatinib
ITGA5	5	--	0	0.45	Tauroursodeoxycholic acid
GRB2	37	--	0	0.45	Pegademase
JAK3	16	0.45	7.3	0.44	Ruxolitinib, Tofacitinib, Momelotinib, Baricitinib, Fostamatinib, Ritlecitinib, Abrocitinib, Zanubrutinib
P2RY12	1	−0.49	0.49	0.44	Ticlopidine, Treprostinil, Clopidogrel, Promethazine, Epoprostenol, Prasugrel, Cangrelor, Ticagrelor
BTK	19	−0.271	5.08	0.44	Dasatinib, Ibrutinib, Acalabrutinib, Fostamatinib, Ritlecitinib, Zanubrutinib, Pirtobrutinib
PPIA	1	--	0	0.44	Cyclosporine, Copper, Artenimol
PIK3CB	1	0.13	0.13	0.44	Caffeine, Copanlisib
CSK	18	0.11	1.95	0.44	Dasatinib, Fostamatinib
PTK2B	37	0.18	6.76	0.44	Leflunomide, Fostamatinib

^1^ FDA-approved drugs from Drugbank v5.1.12. ^2^ Only high-score nodes are targeted.

**Table 5:** Key TEP gene expression comparison over four datasets.

Gene	GSE89843 (NSCLC)	GSE183635 (NSCLC)	GSE68086 (Pancreatic Cancer)	GSE183635 (Breast Cancer)	Values
***ITGA2B***	0.728134021	0.4243767	2.073708	0.6252545	**LogFC ^1^**
	1.40 × 10^−29^	2.20 × 10^−4^	1.25 × 10^−13^	1.72 × 10^−3^	**adj.*p*-value ^2^**
	142/14545	3458/14608	2598/14784	407/17851	**DEG ranking ^3^**
***FLNA***	0.808899158	0.354511	3.144132	0.7036141	**LogFC**
	1.22 × 10^−31^	7.92 × 10^−4^	1.43 × 10^−21^	1.48 × 10^−4^	**adj.*p*-value**
	102/14545	5047/14608	409/14784	247/17851	**DEG ranking**
***GRB2***	0.030350283	−0.1527763	1.188885	0.229971	**LogFC**
	Not significant	3.79 × 10^−2^	7.34 × 10^−11^	1.15 × 10^−1^	**adj.*p*-value**
	Not among top	10092/14608	5890/14784	5659/17851	**DEG ranking**
***FCGR2A***	0.786305852	0.8687625	1.745807	0.633244	**LogFC**
	4.03 × 10^−19^	1.21 × 10^−24^	1.62 × 10^−12^	1.95 × 10^−6^	**adj.*p*-value**
	118/14545	159/14608	3523/14784	387/17851	**DEG ranking**
***APP***	0.336303527	0.2051573	1.344015	0.4870745	**LogFC**
	5.93 × 10^−8^	2.23 × 10^−2^	3.48 × 10^−13^	4.25 × 10^−3^	**adj.*p*-value**
	2257/14545	8645/14608	5126/14784	992/17851	**DEG ranking**

^1^ LogFC = logarithm of fold change; ^2^ adj.*p*-value = multiple testing corrected adjusted *p*-value; ^3^ DEG ranking = differential gene expression ranking.

## Data Availability

The data presented in this study are openly available in [ENA] at [https://www.ebi.ac.uk/ena/browser/view/PRJNA353588], reference number [Bioproject ID: PRJNA353588, GSE89843]. Scripts for all the analyses can be found at https://github.com/ozgeosmanoglu/Platelet_NSCLC (accessed on 28 October 2025).

## Supplementary Materials

The following supporting information can be downloaded at https://www.mdpi.com/article/10.3390/ijms262110780/s1. Refs. [181,182,183,184,185,186,187] are cited in the Supplementary Materials.
