# Supplementary material for: Network Controllability Reveals Key Mitigation Points for Tumor-Promoting Signaling in Tumor-Educated Platelets
Source: Int J Mol Sci. 2025 Nov 5;26(21):10780. doi: 10.3390/ijms262110780 (PMC12609506; doi:10.3390/ijms262110780)
Supplement: Supplementary file 1 [file ijms-26-10780-s001.zip › ijms-3906860 Supplementary_proofed.pdf]

Supplementary Text

Platelet network represents key signaling pathways in platelet function

The platelet signaling network, constructed using directed, signed, and quality-filtered interactions from Omnipath, encompasses 962 interactions among 401 platelet specific proteins. Among 401, genes encoding 227 were significantly regulated in NSCLC and 9 of them were DEGs (*EEF1B2, FLNA, ITGA2B, ARL2, MYL9, CA2, FCGR2A, MAPKAPK2, WASF1*).

Pathway overrepresentation analysis revealed 123 non-redundant Reactome Pathways (**Figure S2**). Immune-related pathways, particularly cytokine signaling, along with Rho GTPase signaling, receptor tyrosine kinase signaling, and platelet-specific signaling, are among the most significantly overrepresented pathways. This strong representation of expected platelet pathways validates the network's ability to accurately capture the complexity of platelet signaling.

Within the network, 61 nodes correspond to proteins found in the "Platelet activation, signaling, and aggregation" pathway (R-HSA-76002). Core platelet activation signaling is represented by 55 interactions among 39 of these key nodes, mediated by central hubs such as SRC, PRKCA, SYK, and MAPK1 (**Figure S7A**). These hubs play critical roles in propagating the signaling necessary for platelet activation, while 22 nodes, which were disconnected to the rest of the network, include important platelet proteins like PECAM1, TRPC6, CFL1, RAP1A.

In addition to platelet activation, the network also highlights Rho GTPase signaling, a pathway integral to cytoskeletal dynamics and platelet shape change. The core network for Rho GTPase signaling includes 77 interactions among 52 proteins, with 37 disconnected nodes omitted for clarity. This network is also centered around SRC, a key signaling hub, and includes essential Rho GTPase proteins such as CDC42 and RAC1. Furthermore, it features proteins involved in actin cytoskeleton regulation, like MYL9, MYLK, FLNA, and CTTN, as well as those involved in microtubule dynamics, such as TUBA4A (**Figure S7B**).

Overall, the platelet network represents how complex platelet communication can be and points out important processes and interactions needed for their function.

Drug Combination in Network Controllability

However, for the remaining proteins, we identified potential targeted therapies. Specifically, sodium sulfate (or topiramate, acetazolamide—only minor interaction with minocycline) was selected for CA2, aducanumab for APP, fostamatinib for JAK2 (or pacritinib, momelotinib if a more specific inhibitor is needed), PRKCD (no other drugs available), MAPK14 (or minocycline), CAMK1, and PTK2, and minocycline for MAPK3 (or sulindac), CASP3 (or acetylsalicylic acid), and MAPK1 (or arsenic trioxide – inducer –). Based on this, we propose a combination of fostamatinib, minocycline, sodium sulfate (or topiramate), and aducanumab as a therapeutic strategy covering a broad range of indispensable nodes while avoiding known drug interactions.

Using kinase inhibition data from Rolf et al. [75], we assessed fostamatinib's efficacy. At 10 μM, R406 showed 99% inhibition of JAK2 in the KINOMEscan assay, with a K_d of 0.78 nM and an IC_{50} of 7 nM (confidence interval: 6–8 nM), confirming strong target engagement. PRKCD, on the other hand, was inhibited by only 17% at 10 μM, indicating weak or no significant binding, with no reported K_d or IC50 values. Similarly, MAPK14 showed only 5% inhibition at 10 μM, suggesting it is not a relevant target of R406. In contrast, R406 inhibited CAMK1 by 97% at 10 μM, with a K_d of 550 nM and an IC_{50} of 1190 nM (confidence interval: 860–1650 nM), indicating strong binding and functional inhibition. PTK2 (FAK) was inhibited by 98% at 10 μM, with a K_d of 550 nM and an IC_{50} of 310 nM (confidence interval: 220–450 nM), further confirming its inhibition.

Since minocycline has shown inhibition of caspase-3 and MAP kinases in animal models [181-183] and *in vitro* [184-185] but lacks direct validation in humans [186,187], we considered alternatives. MAPK3 can also be targeted by sulindac, CASP3 by acetylsalicylic acid, and MAPK1 can be induced by arsenic trioxide, though this effect may not be desirable. If minocycline is excluded, acetazolamide can be used as a more specific alternative for CA2, with only minor interaction with minocycline.

Conclusion: We analyzed TEP transcriptome data compared to controls. We leverage network controllability and drug interactions to identify an optimized, clinically relevant multi-target intervention using FDA-approved drugs that could enhance therapeutic outcomes both regarding TEP and cancer progression.

Controlling TEP activity revealed five drugs as optimal (Fostamatinib, Acetazolamide, Aducanumab, Sulindac, Acetylsalicylic acid). To suggest a focused combination therapy, we prioritized the indispensable nodes based on control centrality, edge count, and betweenness centrality. Amyloid-beta precursor protein (APP) and caspase 3 (CASP3) consistently emerged as key targets alongside fostamatinib's targets. Based on this, we refine our proposed combination to fostamatinib (inhibits JAK2, PTK2, CAMK1), aducanumab (inhibits APP), and acetylsalicylic acid (inhibits CASP3) as the safest and most effective combinatorial strategy for modulating platelet activity in cancer while minimizing the complexity of the regimen.

Supplementary Figures

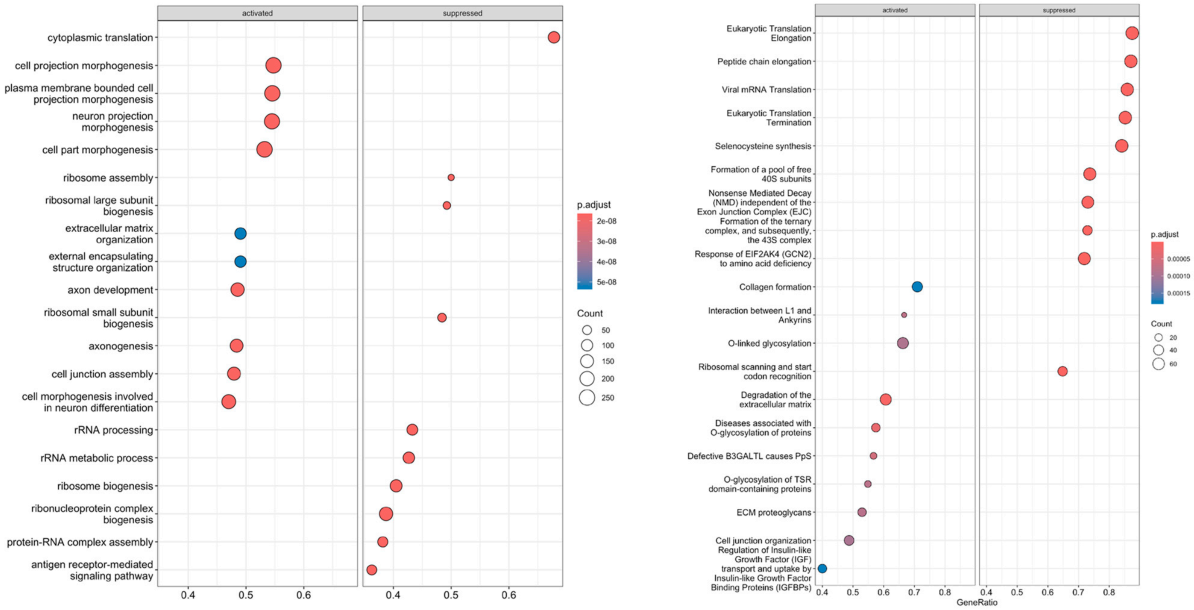

Figure S1: Gene Set Enrichment analysis of NSLCL TEPs (left) Enriched Gene Ontology Biological processes. **(right)** Enriched Reactome pathways.

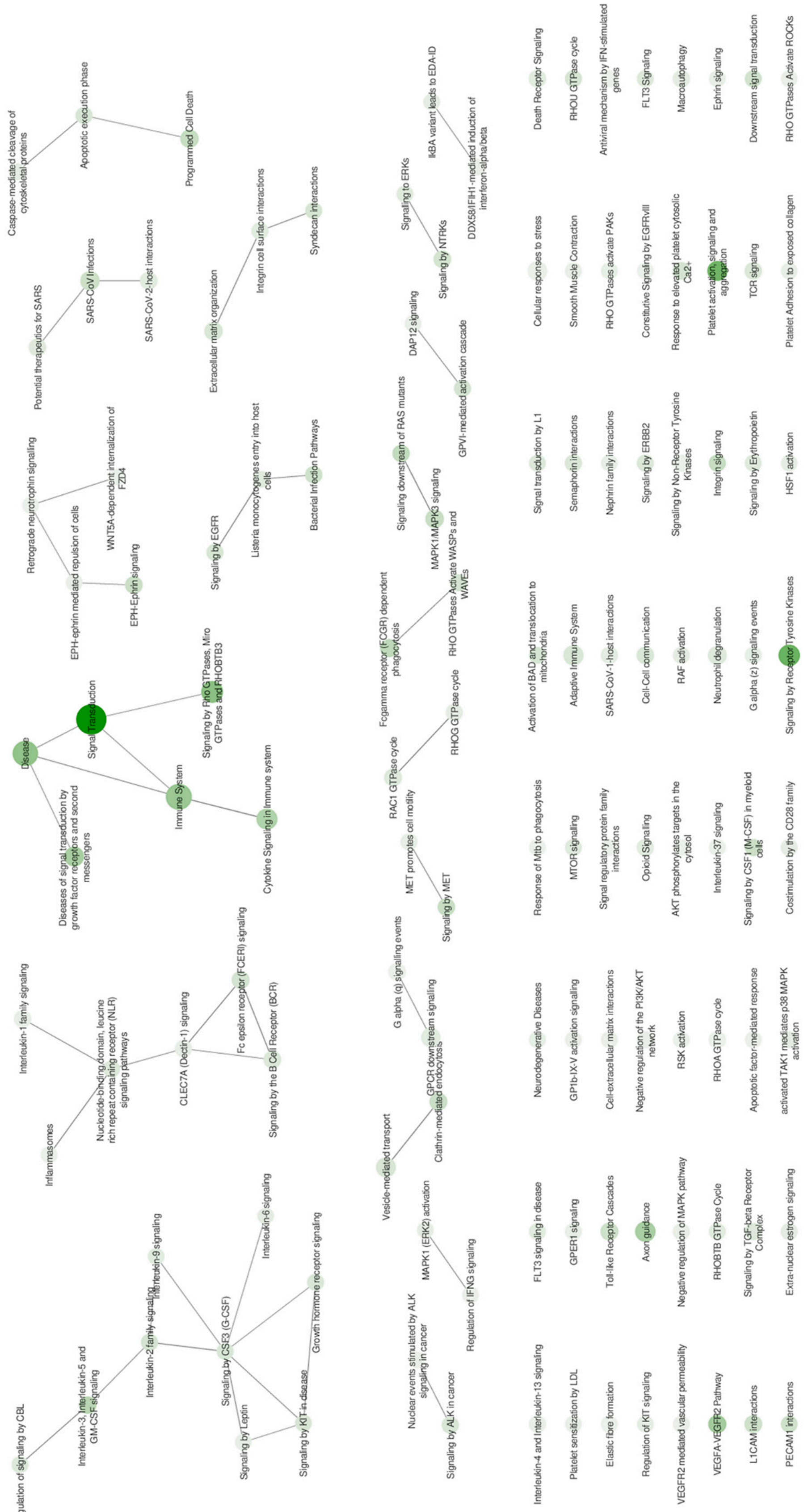

Figure S2: Reactome pathway enrichment in platelet signaling network. All overrepresented pathways in the platelet network. Size increases with increasing term size and color gradient moves towards green with increasing significance (-log10(padj))

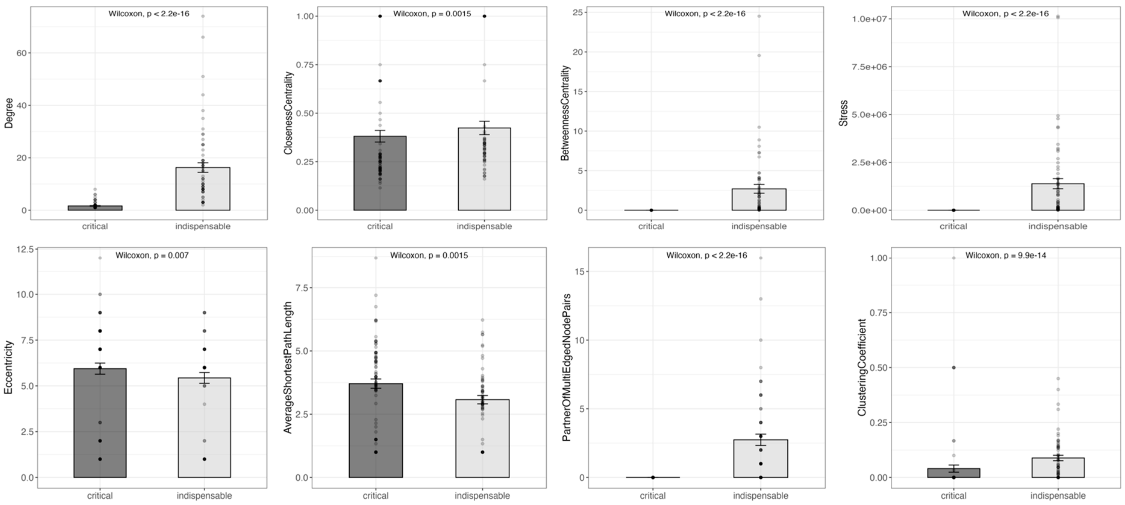

Figure S3: Comparison of topological features of critical and indispensable nodes in the platelet network

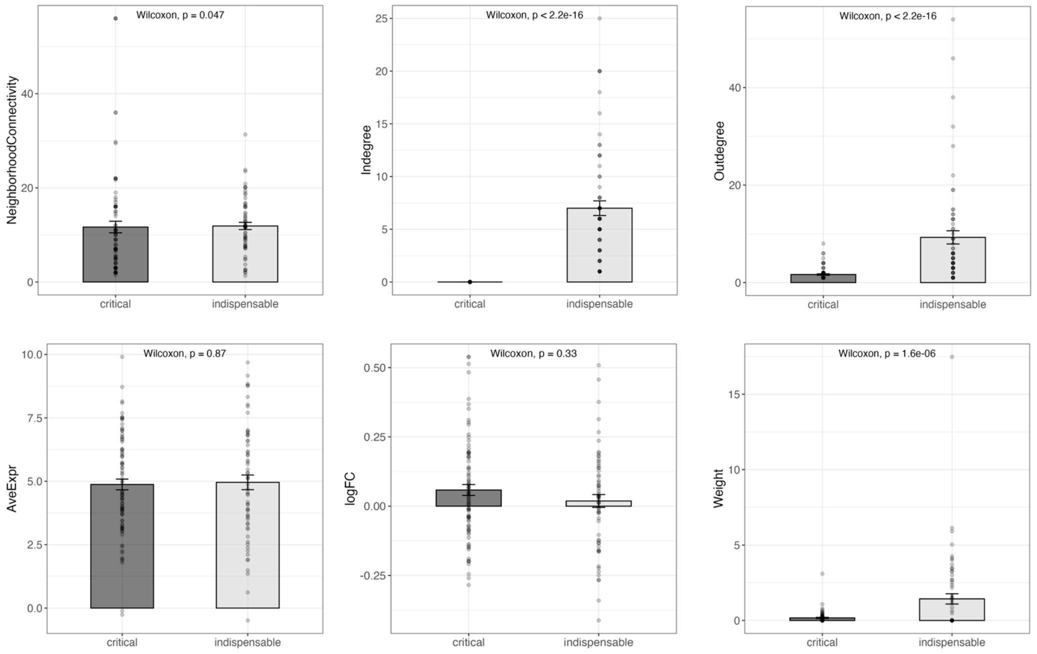

Figure S4: Additional topological and functional features of critical and indispensable nodes

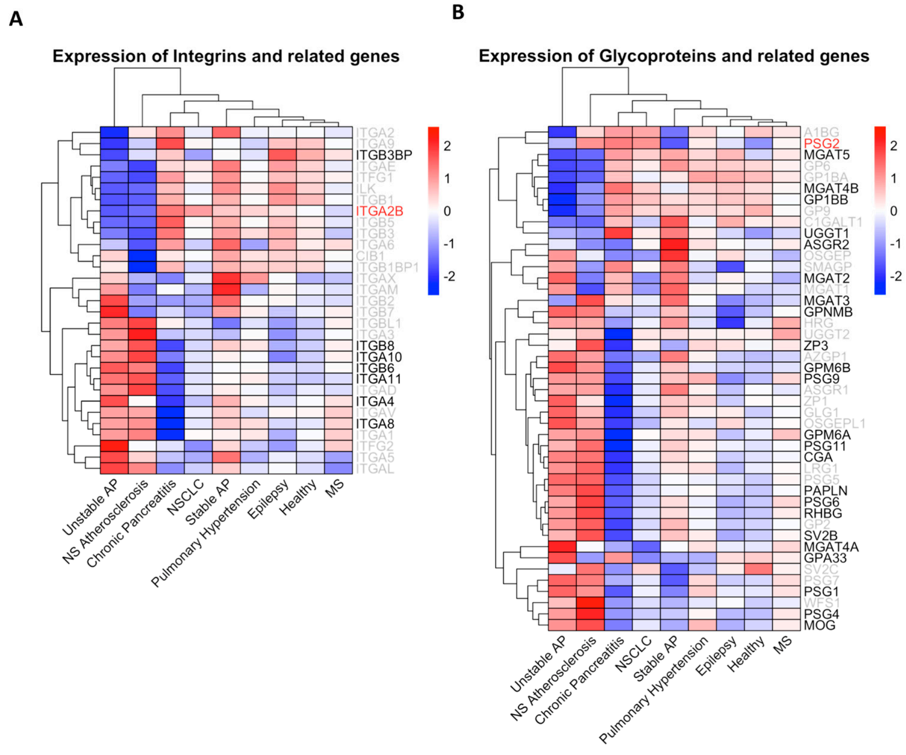

Figure S5: Expression of **(A)** integrins and **(B)** glycoproteins in platelets

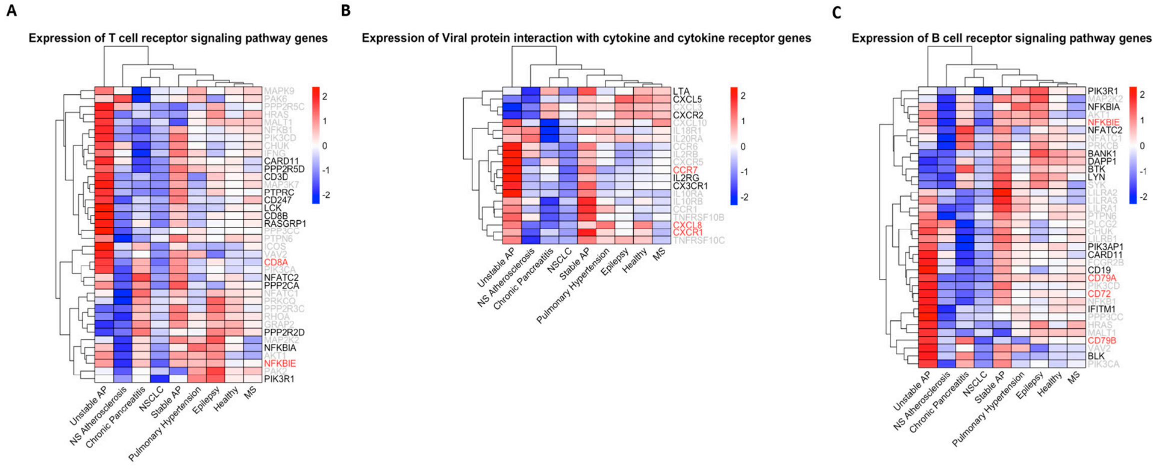

Figure S6: Expression of genes in chosen downregulated KEGG pathways (A) T cell receptor signaling **(B)** Viral protein interaction with cytokine and cytokine receptor genes **(C)** B cell receptor signaling

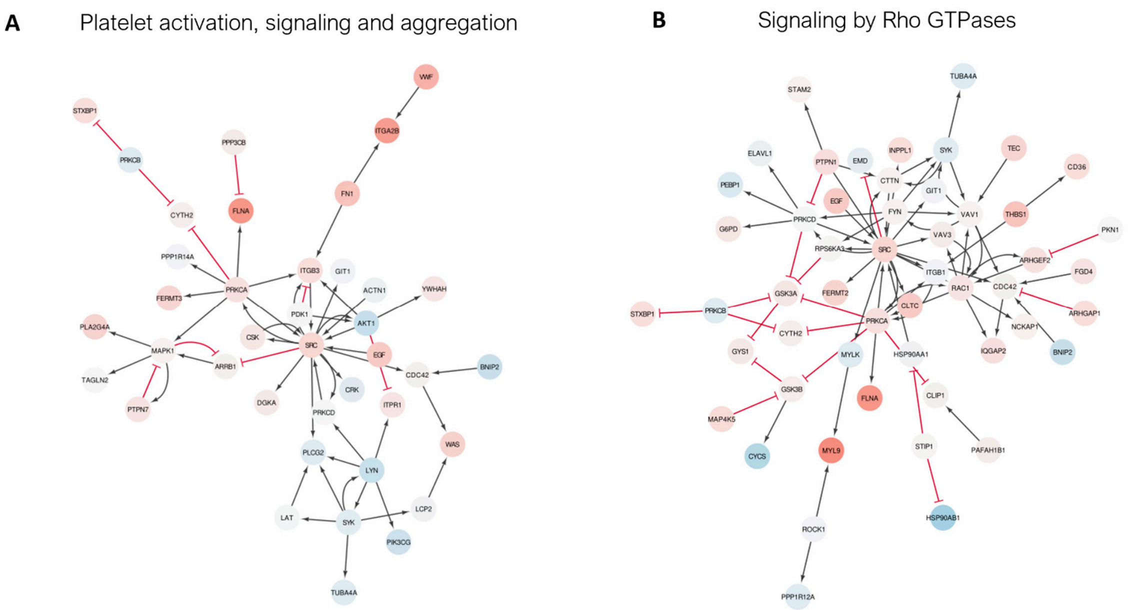

Figure S7: Pathway enrichment in Platelet Signaling Network (A) Platelet activation Reactome pathway **(B)** RhoA signaling Reactome pathway. Red: upregulated, blue: downregulated.

Supplementary Tables

Table S1: Summary of Patient Characteristics. --: not available

	Cancer (n=402)	Non-cancer (n=377)
**Diseases**		
NSCLC	402	--
Healthy Control	--	234
Chronic Pancreatitis	--	6
Non-significant atherosclerosis	--	13
Pulmonary Hypertension	--	34
Unstable Angina Pectoris	--	6
Epilepsy	--	21
Multiple Sclerosis	--	58
Stable Angina Pectoris	--	5
**Metastasis**		
Yes	344	--
No	57	--
NA	1	--
**Gender**		
Female	165	204
Male	189	159
NA	48	14
**Smoking**		
Yes	69	44
Former	153	28
No	88	208
NA	92	97

**Hospital**		
AMC	--	9
HGTP	2	--
MGH	70	--
NKI	150	--
PISA	--	1
UMCU	--	40
UMEA	--	10
VUMC	180	317
**Storage Time**		
< 12h	332	368
> 12h	70	9

Table S2: IGF Pathway drug targets

Target Name[1]	Description	Drugs[2]
APP	Amyloid-beta precursor protein	Deferoxamine,Tromethamine,Copper,Florbetaben F-18,Florbetapir (18F),Flutemetamol (18F),Aducanumab,Zinc acetate,Zinc chloride,Lecanemab
CP	Ceruloplasmin	Zinc acetate, Ferrous fumarate, Zinc chloride
F5	Coagulation factor V	Drotrecogin alfa, Thrombin, Protein C, Human thrombin, Thrombin alfa, Protein S human, Anti-inhibitor coagulant complex
FN1	Fibronectin	Ocriplasmin, Zinc acetate, Zinc chloride
ITIH2	Inter-alpha-trypsin inhibitor heavy chain H2	Copper, Zinc acetate, Zinc chloride
PLG	Plasminogen	Alteplase, Urokinase, Reteplase, Tenecteplase, Aminocaproic acid, Aprotinin, Copper
SERPIND1	Heparin cofactor 2	Copper, Zinc acetate, Zinc chloride

[1]FDA approved drugs from Drugbank v5.1.12.
[2]Only insulin pathway nodes are targeted.

Table S3: Top 10 high weight nodes in platelet interactome

Uniprot ID	Name	Degree	log2FC	Weight[1]	Gene Score
P12931	SRC	134	0.24	31.65	0.43
P17252	PRKCA	84	0.16	13.07	0.40
P07948	LYN	48	−0.24	11.34	0.48
P42224	STAT1	19	−0.51	9.74	0.39
P27986	PIK3R1	28	−0.34	9.53	0.45
P49137	MAPKAPK2	14	0.63	8.78	0.36
Q16539	MAPK14	50	0.16	8.12	0.35
P27361	MAPK3	55	0.15	8.07	0.39
P52333	JAK3	16	0.46	7.30	0.44
Q14289	PTK2B	37	0.18	6.76	0.44

[1] Weights are calculated by: W(i) = |log2FC(i)| x Degree(i)

Table S4: Top 10 high score nodes in platelet interactome

Uniprot ID	Name	Degree	log2FC	Weight	Gene Score
Q92835	INPP5D	4	—	0.00	0.51
P16885	PLCG2	8	—	0.00	0.48
P22681	CBL	29	0.20	5.67	0.48
P07948	LYN	48	−0.24	11.34	0.48
P02730	SLC4A1	3	0.54	1.62	0.48
Q96B97	SH3KBP1	2	—	0.00	0.47
Q15642	TRIP10	1	−0.26	0.26	0.47
Q8NDB2	BANK1	1	−0.25	0.25	0.47
Q9UN19	DAPP1	5	−0.41	2.04	0.47
P23458	JAK1	21	—	0.00	0.47

References

For the detailed information on the references cited here, please see the main text references section.
